# The Factor Inhibiting HIF Asparaginyl Hydroxylase Regulates Oxidative Metabolism and Accelerates Metabolic Adaptation to Hypoxia

**DOI:** 10.1016/j.cmet.2018.02.020

**Published:** 2018-04-03

**Authors:** Jingwei Sim, Andrew S. Cowburn, Asis Palazon, Basetti Madhu, Petros A. Tyrakis, David Macías, David M. Bargiela, Sandra Pietsch, Michael Gralla, Colin E. Evans, Thaksaon Kittipassorn, Yu C.J. Chey, Cristina M. Branco, Helene Rundqvist, Daniel J. Peet, Randall S. Johnson

**Affiliations:** 1 Physiological Laboratory, Department of Physiology, Development and Neuroscience, University of Cambridge, Cambridge CB2 3EG, UK; 2 Department of Cell and Molecular Biology, Karolinska Institute, Stockholm SE-171 77, Sweden; 3 Cancer Research UK Cambridge Institute, Cambridge CB2 0RE, UK; 4 School of Biological Sciences, University of Adelaide, Adelaide, SA 5005, Australia; 5 Department of Physiology, Faculty of Medicine, Siriraj Hospital, Mahidol University, Bangkok 73170, Thailand

**Keywords:** hypoxia, metabolism, HIF

## Abstract

Animals require an immediate response to oxygen availability to allow rapid shifts between oxidative and glycolytic metabolism. These metabolic shifts are highly regulated by the HIF transcription factor. The factor inhibiting HIF (FIH) is an asparaginyl hydroxylase that controls HIF transcriptional activity in an oxygen-dependent manner. We show here that FIH loss increases oxidative metabolism, while also increasing glycolytic capacity, and that this gives rise to an increase in oxygen consumption. We further show that the loss of FIH acts to accelerate the cellular metabolic response to hypoxia. Skeletal muscle expresses 50-fold higher levels of FIH than other tissues: we analyzed skeletal muscle FIH mutants and found a decreased metabolic efficiency, correlated with an increased oxidative rate and an increased rate of hypoxic response. We find that FIH, through its regulation of oxidation, acts in concert with the PHD/vHL pathway to accelerate HIF-mediated metabolic responses to hypoxia.

## Highlights

• FIH loss increases oxidative metabolism, while also increasing glycolytic capacity
• This accelerates the hypoxic response, ultimately suppressing oxidative metabolism
• FIH acts in concert with PHD/vHL to accelerate HIF-mediated responses to hypoxia
• This rapid adaption serves tissues with high levels of oxygen flux, such as muscle

## Graphical Abstract

**Figure:**
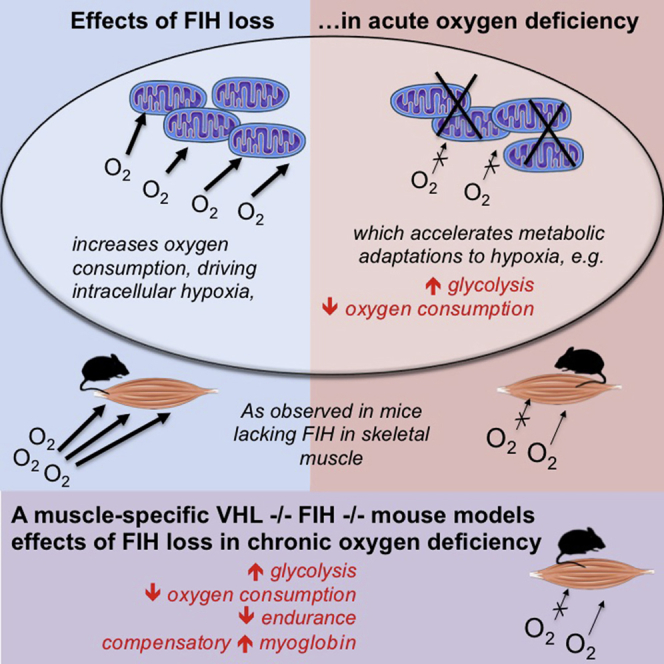

## Teaser

Cells transition between aerobic and anaerobic metabolism when adapting to changes in oxygen supply. Sim et al. find that the enzyme FIH is expressed at high levels in skeletal muscle and decreases in activity under hypoxia. With the loss of FIH, cells have increased aerobic metabolism, which paradoxically accelerates other adaptations to hypoxia.

## Introduction

In eukaryotes, oxygen is the terminal electron acceptor in respiration and is essential for the synthesis of both cellular machinery and signaling molecules. Hypoxia-inducible factors (HIFs) are key regulators of the transcriptional response to shifts in oxygenation (Semenza and Wang, 1992). Many metabolic pathologies arise from inappropriate changes in HIF activity (Girgis et al., 2012), underscoring the need for regulation of HIF function.

The activity of the heterodimeric HIF transcription factor complex depends on both the abundance (Wang and Semenza, 1993) and the transactivational capacity of the alpha subunits (HIF-α) (Mahon et al., 2001). Their abundance is regulated by prolyl hydroxylases (PHDs) 1–3, which hydroxylate proline residues on HIF-α (Epstein et al., 2001), enabling the von Hippel-Lindau (vHL) ubiquitin ligase complex to target HIF-α (Jaakkola et al., 2001) for proteasomal degradation (Maxwell et al., 1999). Oxygen is absolutely required for hydroxylase activity. As oxygen levels drop, HIF-α escapes vHL-mediated degradation and accumulates (Min et al., 2002).

HIF-α transactivational capacity is controlled by an asparaginyl hydroxylase (factor inhibiting HIF, or FIH) (HIF-1AN), acting on Asn803 in the C-terminal domain (C-TAD) of the HIF-1α protein. The hydroxylation of this residue prevents HIF from recruiting the transcriptional coactivator and histone acetyltransferase p300/CBP to the HIF-α C-TAD (Dames et al., 2002, Lando et al., 2002). Repression of FIH activity under hypoxia is both necessary and sufficient for disinhibition of HIF-1α C-TAD activity (Lando et al., 2002, McNeill et al., 2002, Zhang et al., 2010). While there are three major PHD isoforms, there is only one known FIH isoform (Elkins et al., 2003, Mahon et al., 2001), and in its absence, no hydroxylation of the Asn803 residue occurs (Zhang et al., 2010).

FIH and the PHDs share some enzymatic properties, including cofactors and by-products (Hewitson et al., 2002); this has led to the assumption that PHDs and FIH are functionally redundant in their regulation of HIF. It has also been difficult to account for FIH's distinct evolutionary history as an oxygen sensor (Hampton-Smith and Peet, 2009, Taylor and McElwain, 2010): FIH was present in the very earliest stages of the evolution of animal oxygen sensing, but is absent in a few intermediate forms, including some arthropods, while preserved in most others, including all vertebrates.

An interesting dilemma in understanding the role of FIH contra the PHD/vHL pathway is that FIH has a lower K_m_ for oxygen than the PHD enzymes (Ehrismann et al., 2007, Tarhonskaya et al., 2015). An additional complexity is the data that indicate that the HIF-1α isoform is more susceptible to FIH modification than the other major HIF isoform, HIF-2α (Bracken et al., 2006, Koivunen et al., 2004). HIF-1α is the HIF-α isoform that directly regulates expression of many of the enzymes that control metabolism (Keith et al., 2012); thus, a differential control of HIF-1α by FIH could potentially trigger specific shifts in metabolic response. However, the fact that FIH is still an active hydroxylase at lower oxygen levels than those that would trigger HIF accumulation indicates that FIH could be particularly relevant where a rapid onset of hypoxia outstrips the process of HIF-1α accumulation (by PHD inhibition). In other words, the FIH pathway could serve as a failsafe for when an inappropriate, relative deficiency of HIF places the cell at a metabolic disadvantage.

We have shown that FIH nullizygous mice have an increased lean muscle mass and an increased mass-specific VO_2_ (Zhang et al., 2010). This was surprising, as other data showed that HIF-α overexpression via loss of vHL or PHD1 results in *decreased* oxygen consumption in cells (Aragones et al., 2008, Fukuda et al., 2007, Kim et al., 2006, Papandreou et al., 2006, Zhang et al., 2008) and animals (Yaqoob and Schwerte, 2010). Animals with HIF overexpression via the PHD/vHL pathway also show impaired aerobic exercise capacity (Aragones et al., 2008, Formenti et al., 2010, McClain et al., 2013), despite increased muscle capillarization (Karsikas et al., 2016, Lijkwan et al., 2014).

The roles of FIH in cellular metabolism have thus to date been unclear. Hypoxic cells express a preference for anaerobic metabolism, which can lead to a catabolic state (Frezza et al., 2011) depending on the cell's nutrient status. Indeed, pan-PHD deletion (Duan et al., 2014), and singular PHD1 (Aragones et al., 2008), PHD2 (Minamishima et al., 2009), or vHL loss (Hervouet et al., 2005, Wise et al., 2011, Zhang et al., 2007) all give rise to the classical cellular response to hypoxia, i.e., decreased mitochondrial activity, increased glycolysis, and glycogen and lipid accumulation.

In this study, we demonstrate that FIH has a specific role in the control of metabolism, a role essential for potentiation of metabolic responses to shifts in oxygenation. This role diverges from the role of the PHD/vHL pathway, acting to accelerate the rate of oxygen consumption, and we propose that this can increase the rapidity and magnitude of the hypoxic response.

## Results

### Quantitative Effects of FIH Loss on the Metabolic Transcriptome

**Figure 1:**
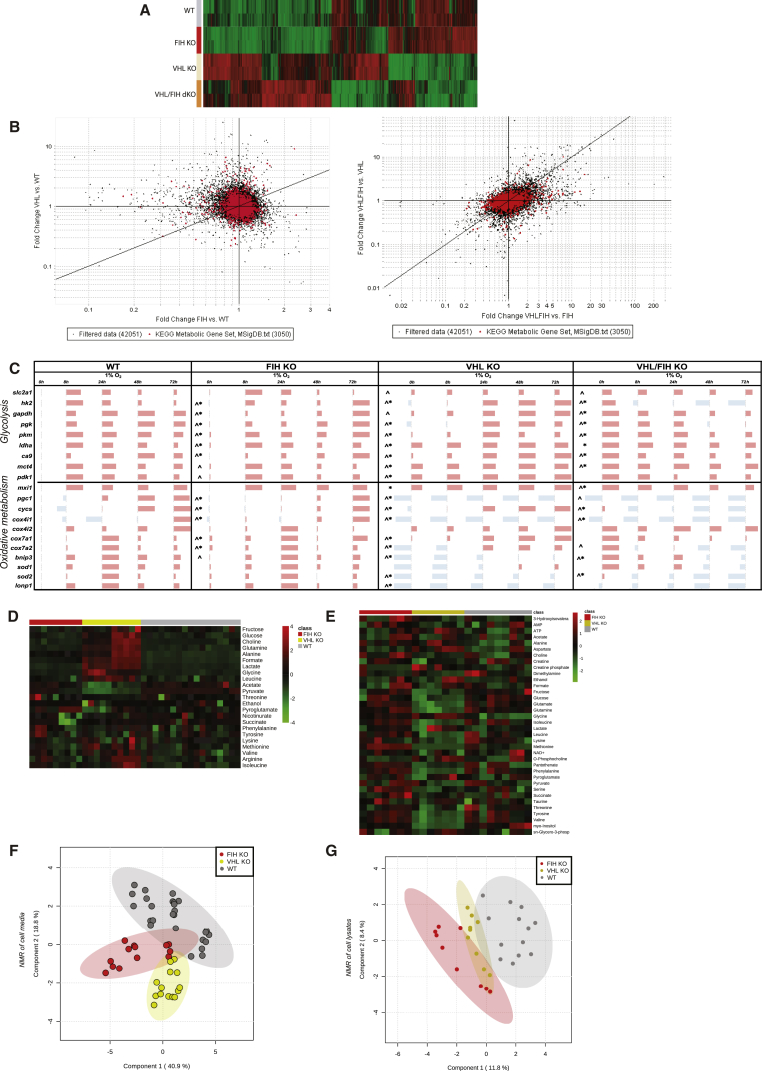
FIH Is a Non-redundant Regulator of Metabolic Parameters and Metabolic Gene Expression (A) Heatmap analysis of microarray data: each row denotes a sample, while each column denotes a gene transcript; net fold changes in gene expression are normalized to column means. Red indicates that a transcript has been significantly upregulated relative to the column mean; green indicates downregulation. A total of 5,000 genes that varied the most with genotype are depicted here. (B) Scatterplot analysis of microarray data: fold change in gene expression that results from acute FIH versus vHL deletion in MEFs. Each data point represents an mRNA transcript. Fold change expression following FIH loss (x axis), and fold change expression following vHL loss (y axis) for the first plot, and the effect of concomitantly knocking out vHL and FIH together compared with single FIH loss (x axis), and compared with single vHL loss (y axis) for the second plot. Genes with metabolic Kyoto Encyclopedia of Genes and Genomes annotations are highlighted in red. (C) qRT-PCR analysis of control MEFs and KO MEFs. Dark red shading indicates an upregulation of the gene transcript relative to control MEFs at 0 hr; light blue shading indicates a downregulation. A two-way ANOVA analysis was performed, to dissect the contributions of genotype and time to expression. ˆ Denotes a significant interaction between time exposed to hypoxia and genotype (p < 0.0001) on gene expression, while ^∗^ denotes that genotype alone has a significant effect on gene expression (p < 0.0001). The leftmost column in each section reflects genotypic comparisons between normoxic cells, whereas the other data refer to the effect of genotype and indicated duration of hypoxic exposure. (D) Heatmap analysis of ^1^H-nuclear magnetic resonance (NMR) data. Red indicates that a metabolite has been significantly upregulated relative to the row mean, while green indicates downregulation. Each column denotes an independent cell culture sample. Only aqueous metabolites with the highest absolute abundance are shown. (E) Heatmap of ^1^H-NMR data performed in MetaboAnalyst: aqueous metabolites in whole MEF lysates. (F) Principal component analysis (PCA) of ^1^H-NMR data performed in MetaboAnalyst: each data point denotes an independent cell culture media sample. Each genotype is demarcated by a 95% confidence interval (oval). (G) PCA of ^1^H-NMR data performed in MetaboAnalyst: each data point denotes an independent sample of whole-cell lysates. See also Figure S1.

Microarray analysis of an FIH/vHL null cell dataset (GEO: GSE20335) (Figure 1A) from mRNA derived from murine embryonic fibroblasts (MEFs) (Figure S1A) under normoxic culture shows that FIH loss affects the transcriptome differently than vHL loss. In an analysis of individual gene changes, FIH is able to act both as an inducer and a suppressor of a variety of genes, including genes that have Kyoto Encyclopedia of Genes and Genomes annotations in metabolic pathways (Figure 1B), and there is a clear differentiation between the effects of FIH deletion and vHL deletion across the metabolic transcriptome. Deletion of both factors, as in the broader transcriptome, has differentiable effects from either single deletion.

In Figure 1B, there appears to be no relation between fold change in gene expression in vHL null cells and fold change in FIH null cells (left panel), but a reasonable correlation between the effect of FIH inactivation in vHL null cells and that of vHL inactivation in FIH null cells (right panel). One explanation would be that FIH and vHL have concordant effects in the presence of large amounts of HIF protein, but that FIH has additional or different actions when HIF levels are low. It has been noted that the N termini of FIH and vHL interact with HIF-1α at distinct sites (Mahon et al., 2001) to recruit different HDACs. When HIF-1α is abundant, it could scaffold the interaction of FIH and vHL, such that the inactivation of either influences the other.

### Metabolic Differences in FIH and vHL Null Cells

To determine how hypoxia affects FIH regulation of metabolic genes, a qRT-PCR study of selected transcripts in MEFs was carried out. We found that FIH acting alone in cells at 21% oxygen levels again differentiates these two post-translational regulators of HIF expression (Figure 1C). At 0 hr (normoxia), vHL loss increases expression of most glycolytic genes, while causing decreased levels of a range of genes controlling oxidative metabolism. These changes in expression continue into the early time points of hypoxic (1% oxygen) conditioning. FIH loss increases glycolytic gene expression over 8–72 hr of hypoxia, but clamps oxidative gene expression in normoxia and early hypoxia, rather than suppressing it. Note specifically that there are significant differences in FIH and vHL regulation of cox4i1/2 and cox7i1/2 transcripts; their expression is known to be sensitive to prevailing oxygen levels (Fukuda et al., 2007, Hwang et al., 2015). Subunit 4, in particular, is rate-limiting for complex IV assembly and function, with implications for cell survival and ATP levels in hypoxia (Li et al., 2006).

To determine how these gene expression changes act on cellular metabolism, a ^1^H-nuclear magnetic resonance metabolomics analysis was performed on extracellular metabolites from media samples following 48 hr of cell culture (Figure 1D; Tables S2 and S3), and on cell lysates (Figure 1E). There is a clear separation of FIH null, vHL null, and control aqueous metabolite profiles, with lactate, alanine, glucose, and glutamine showing significant fold changes (Figure 1F). Intracellular aqueous metabolites of FIH null and control cells were more widely separated (Figure 1G) than between vHL null and control cells, indicating significant and distinct metabolic differences in cells with and without FIH.

### FIH Modulates a Hypoxic Metabolic Shift

**Figure 2:**
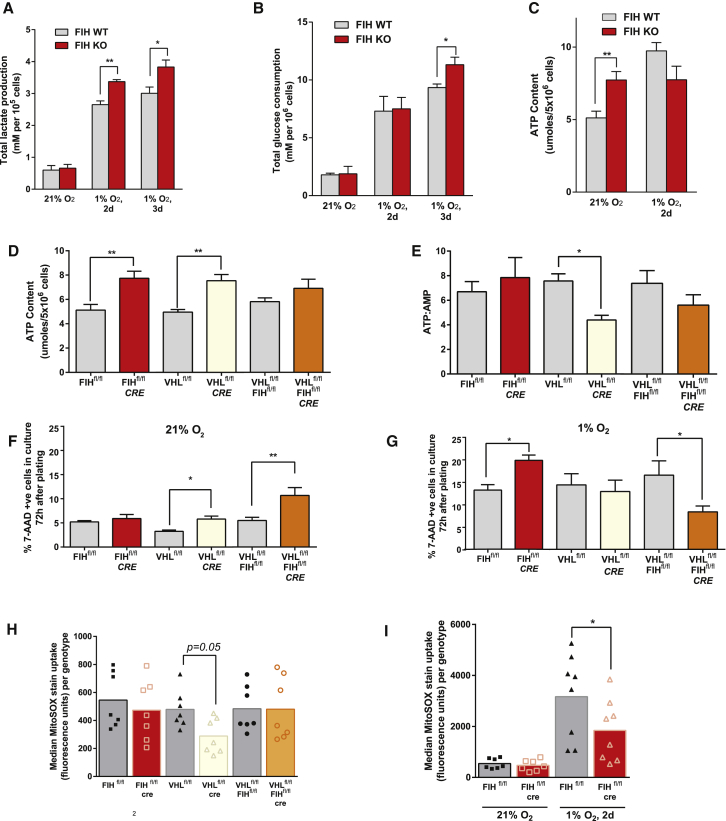
FIH Loss Promotes Glycolysis in Hypoxia, but Not Normoxia (A) Enzyme-based colorimetric assay of culture media lactate. (B) Enzyme-based colorimetric assay of culture media glucose. (C) HPLC-based ATP measurements from cell lysates. (D) HPLC-based ATP measurements from cell lysates. (E) AMP:ATP ratio in cell lysates. (F) 7′-AAD flow cytometric apoptosis assay for cells cultured in atmospheric oxygen. (G) 7′-AAD flow cytometric apoptosis assay for cells cultured in 1% oxygen for 3 days. (H) Flow cytometry; histogram of cell count versus MitoSOX (superoxide stain) fluorescence, with shaded graph representing unstained controls. Three experiments were performed with two biological replicates each; the histogram provided is representative of one of these experiments. (I) Flow cytometry; histogram of cell count versus MitoSOX fluorescence, with shaded graph representing unstained controls. ^∗^p < 0.05, ^∗∗^p < 0.01. Data are represented as means ± SEM. For grouped data (A–C), a two-way ANOVA was used. For multiple comparisons (D–G), a one-way ANOVA was used. For pairwise comparisons (H and I), a two-tailed Student's t test was used. n = 3 independent cell culture samples per genotype, unless otherwise specified. See also Figure S2.

vHL and vHL/FIH double null MEFs show increases in lactate production and glucose uptake relative to wild-type (WT) cells, but FIH null cells do not (Figures S2A and S2B). However, loss of FIH increases both lactate production and glucose uptake by these cells during prolonged exposure to 1% oxygen over 72 hr (Figures 2A and 2B). This indicates that FIH has a role in modulating hypoxic response even at levels of oxygen as low as 1%, in keeping with the high oxygen affinity of the FIH enzyme. Elevated ATP levels seen in FIH null cells in normoxia (Figure 2C) are lost after 48 hr of exposure to hypoxia.

In sum, the loss of FIH increases both lactate production and glucose uptake over prolonged exposure to 1% O_2_, but not under 21% O_2_. Nevertheless, in Figure 2C, FIH knockout (KO) MEFs have elevated ATP levels at normoxia. We speculate that this is the result of certain oxygen-dependent, anaplerotic pathways becoming hyperactive when FIH is lost, thus permissive for high ATP levels in the cell (albeit with a normal ATP:AMP ratio, i.e., energy supply/demand is balanced). When oxygen is limiting, these pathways are thrust into glucose dependence, or redirected into lactate-producing pathways.

Interestingly, vHL null cells also show an elevated level of ATP relative to WT MEFs; this is not seen in FIH/vHL null cells (Figure 2D). The overall ratio of ATP:AMP is greatly reduced in vHL null cells, but is not reduced significantly in either FIH null or FIH/vHL null cells (Figure 2E), indicating that, coupled to the higher levels of ATP in vHL null cells, there is also a greater rate of ATP turnover. This indicates that there are likely higher cellular energetic demands in vHL null MEFs, and that these are reduced when FIH is also deleted. These data, taken together, argue for an energetically complementary function of these two negative regulators, a synergy that is acting to balance metabolic function during hypoxic response. Further evidence for this comes from cell survival rates: under normoxia, vHL null MEFs have an increased rate of apoptosis, and FIH/vHL null MEFs have an even higher rate of cell death (Figure 2F), whereas the opposite is the case in hypoxia, where FIH null MEFs have a higher rate of apoptosis, and FIH/vHL null MEFs have a significant survival advantage (Figure 2G).

### FIH Suppresses Hypoxia-Induced Mitochondrial Reactive Oxygen Species Production

Key aspects of hypoxic adaptation include a shift of cytochrome oxidase subunits (Aras et al., 2013, Fukuda et al., 2007) and the induction of superoxide dismutase (Rasbach et al., 2010, Scortegagna et al., 2003), both limiting the production of reactive oxygen species (ROS). Loss of FIH does not affect mitochondrial superoxide production at normoxia, but loss of vHL results in a reduction of superoxide production, as measured by the MitoSOX assay (Figures 2H and S2C). Interestingly, further loss of FIH eliminates this difference in ROS production seen in vHL null MEFs, arguing that there is a compensating shift in ROS handling caused by the double deletion. Prolonged culture in hypoxia causes a marked increase in ROS production in WT MEFs (Figures 2I and S2D). However, this increase in ROS under hypoxia is significantly suppressed by the loss of FIH, again indicating that FIH is regulating mitochondrial activity, and indicating as well that native FIH activity has not been completely suppressed at 1% oxygen, even after 2 days of hypoxic culture.

### Shift in Mitochondrial Membrane Potentials Is Induced by vHL Loss, but Not FIH Loss

**Figure 3:**
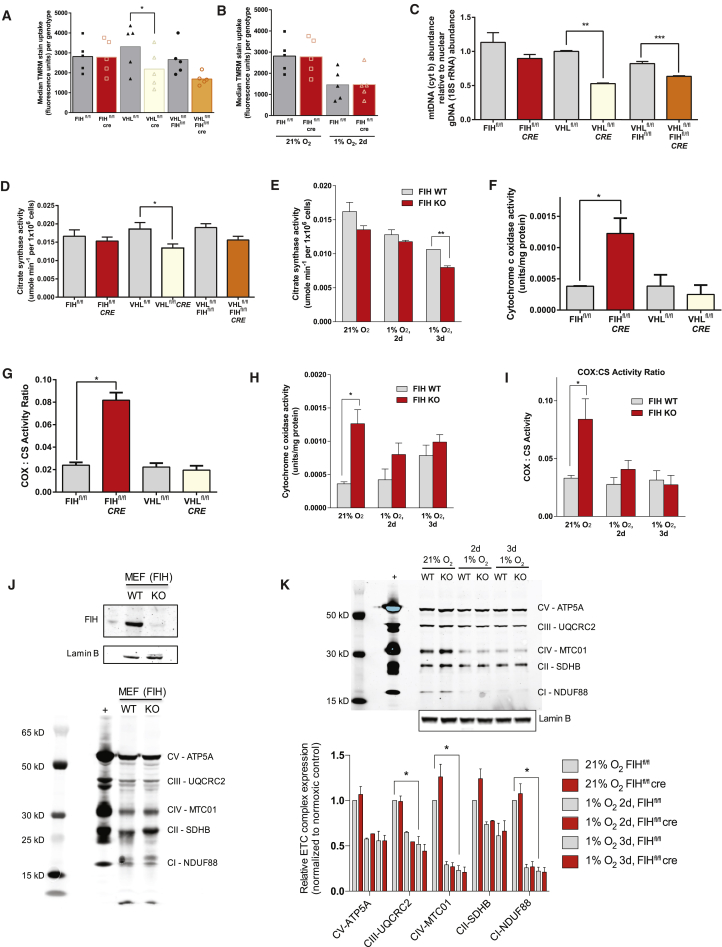
Differential Regulation of HIF Leads to Differential Regulation of Mitochondrial Parameters (A) Flow cytometry; histogram of cell count versus TMRM fluorescence, with shaded graph representing unstained controls. Three experiments were performed with two biological replicates each; this series of histograms is representative of one of these experiments. (B) Flow cytometry; histogram of cell count versus TMRM fluorescence, with shaded graph representing unstained controls. Three experiments were performed with two biological replicates each; this pair of histograms is representative of one of these experiments. (C) mtDNA abundance in indicated cells. (D) Citrate synthase activity measured in indicated cells. (E) Citrate synthase activity in FIH KO compared with control MEFs over prolonged exposure to hypoxia. (F) FIH, but not vHL KO cell lysates show higher mitochondrial cytochrome *c* oxidase activity (p = 0.049) than control MEFs. (G) Cytochrome oxidase activity of FIH KO MEFs normalized to citrate synthase activity. (H) FIH KO MEFs have higher cytochrome oxidase activity than control cells in atmospheric oxygen, but this effect is blunted over hypoxic exposure. (I) The trends described in (H) persist even when the cytochrome oxidase activity of FIH KO and control MEFs is normalized to citrate synthase activity levels. (J) Western blot of control versus FIH KO MEFs with lamin B as a loading control and rat heart mitochondria as a positive control; demonstrates total electron transport chain complex content in MEF lysates. (K) Western blot of mitochondrial complex content in control versus FIH KO MEFs, with progressive hypoxic exposure. ^∗^p < 0.05, ^∗∗^p < 0.01, ^∗∗∗^p < 0.001. Data are represented as means ± SEM. For pairwise comparisons (A and B), a two-tailed Student's t test was used. For multiple comparisons (C, D, F, and G), a one-way ANOVA was used. For grouped data (E, H, I, and K), a two-way ANOVA was used. n = 3 independent cell culture samples per genotype, unless otherwise specified.

The increases in apoptosis in FIH null MEFs in hypoxia, and in vHL and FIH/vHL null MEFs in normoxia, as well as changes induced in mitochondrial ROS production, indicate that FIH may play a role in regulating mitochondrial energetics that is separable from the role played by vHL. As seen in Figures 3A and S2E, loss of vHL significantly decreases mitochondrial membrane potential in normoxia; loss of both FIH and vHL phenocopies loss of vHL alone. This indicates that the PHD/vHL axis has primary control over mitochondrial potential, unlike FIH, which has little effect on mitochondrial potential in either normoxia or hypoxia (Figures 3B and S2F). Thus, FIH modulates ROS production without an effect on mitochondrial potential.

### Mitochondrial Levels and Activity Differ in FIH and vHL Null MEFs

There is a significant reduction in mtDNA in both vHL and FIH/vHL null MEFs, with an almost 50% reduction in vHL null cells (Figure 3C). This correlates with an overall reduction in citrate synthase activity per cell in the vHL null cells (Figure 3D), with no corresponding change in FIH null MEFs. In addition, culture of FIH null MEFs in hypoxia accelerates the loss of mitochondrial citrate synthase relative to the decline seen in WT cells (Figure 3E). This demonstrates that FIH loss accelerates the effects of hypoxic exposure on mitochondria.

### Loss of FIH Increases Mitochondrial Activity under Normoxia

The data above demonstrate the role of FIH in preserving metabolic hypoxic response when some oxygen is still available, e.g., at 1% oxygen, and indicates that complete loss of FIH function accelerates a shift both to a glycolytic metabolism and to a reduced level of mitochondrial functioning.

There is a tripling of cytochrome *c* oxidase (COX) activity in FIH KOs relative to WT cells (Figure 3F). Normalized to citrate synthase activity, and thus to mitochondrial density, we see an almost 4-fold increase in COX activity as a function of mitochondrial content (Figure 3G), which is reduced over 48 hr of exposure to hypoxia (Figure 3H), and lessened as a function of mitochondrial density (Figure 3I). This indicates that the loss of FIH function drives an increase in oxidative metabolism, although this is subsequently lost as hypoxia drives additional adaptations. The loss of FIH drives an increase in electron transport chain components complex II and IV at baseline (Figure 3J), despite the preservation of mitochondrial content shown earlier. Interestingly, in parallel with the loss of mitochondrial activity with hypoxia, the differences in complex II/IV expression between WT and KO cells diminish with prolonged exposure to hypoxia (Figure 3K).

### Loss of FIH under Normoxia Selectively Increases Glycolytic Reserve

**Figure 4:**
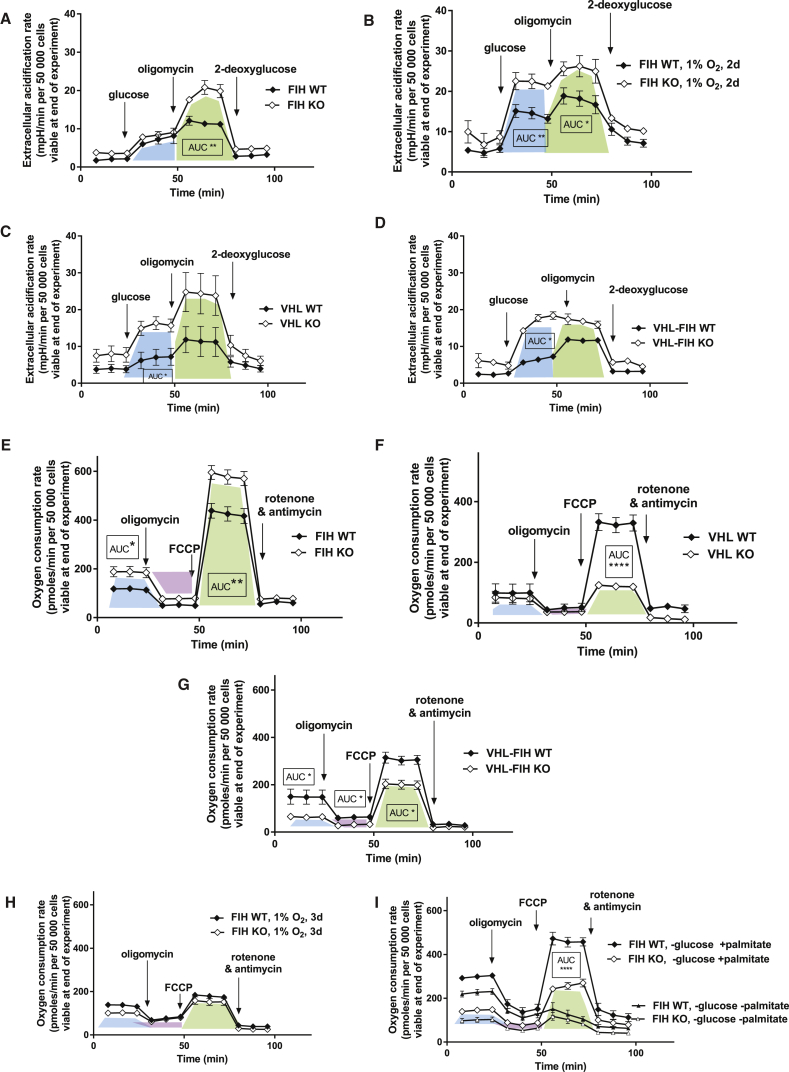
FIH Specifically Determines Glycolytic Reserve and Uncoupled Oxygen Consumption (A) Glycolytic stress test on FIH KO MEFs in 21% oxygen. The area under the curve (AUC) for each genotype was compared with a two-tailed t test. The blue polygon represents the response to glucose supplementation after a period of glucose starvation, i.e., baseline glycolysis. The green polygon is proportionate to the glycolytic reserve, unmasked upon ATP synthase inhibition. (B) Glycolytic stress test repeated on FIH KO MEFs under 1% oxygen after 3 days in hypoxic culture. (C) Glycolytic stress test, on vHL-KO MEFs in 21% oxygen. (D) Glycolytic stress test on vHL-FIH double-KO MEFs. (E) Oxidative stress test. The AUC for each genotype was compared with a two-tailed t test. The blue polygon represents baseline oxygen consumption. The purple polygon is proportionate to ATP-coupled oxygen consumption. The green polygon is proportionate to uncoupled oxygen consumption, unmasked on FCCP addition. (F) Oxidative stress test. Uncoupled VO_2_ is in contrast reduced in vHL KO MEF populations in normoxia. (G) Oxidative stress test. vHL/FIH double-KO MEFs resemble vHL KO MEFs in that they also have reduced uncoupled VO_2_ values. (H) Oxidative stress test repeated under 1% oxygen after 3 days of hypoxic culture. (I) Modified oxidative stress test using a glucose-depleted, palmitate-abundant substrate medium. ^∗^p < 0.05, ^∗∗^p < 0.01, ^∗∗∗∗^p < 0.0001. OCR and ECAR data are represented as means ± SEM. n = 3 independent cell culture samples per genotype. The AUC for every three readings (represented by a single polygon) was computed, and a two-tailed Student's t test was applied for each pair of AUCs compared. See also Figure S3.

To better map metabolic cellular responses of the two HIF post-translational modification mutants, we analyzed proton generation and oxygen consumption in KO MEFs. Loss of FIH does not change acidification rates relative to WT cells, demonstrating that glycolytic rates in these cells are normal; however, the glycolytic reserve is increased significantly (Figure 4A). This indicates that loss of FIH can in some regard metabolically prepare cells for hypoxic glycolytic metabolism. Further evidence of this is seen in Figure 4B, where hypoxic FIH null cells have increased glycolytic rates in addition to a preserved increase in glycolytic reserve. This is compared with the changes in vHL null cells (Figure 4C), which demonstrate increases in both glycolytic reserves and rates typically seen when the PHD/vHL axis is suppressed, or when HIF-1α is overexpressed. Interestingly, although the FIH/vHL KO cells are more glycolytic relative to WT cells (Figure 4D), there is no further increase seen when oligomycin is added to inhibit the mitochondrial ATP synthase. Where both FIH and vHL are fully suppressed, glycolytic activity is equivalent to glycolytic capacity, i.e., the cells are maximally glycolytic, deriving energy mostly from glycolysis as opposed to oxidation.

### Loss of FIH under Normoxia Increases Oxygen Consumption

The loss of FIH causes an overall increase in cellular basal respiration (Figure 4E), and this effect is reversed when the FIH gene is restored via transfection to FIH null cells (Figures S3A and S3B). This unexpected result is in contrast to the expected change in cellular oxygen consumption caused by the loss of vHL (Figure 4F). In vHL null cells, there is essentially an elimination of the spare respiratory capacity. This is consistent with previous studies of vHL null cells where mitochondrial mass is decreased (Haase, 2012, Hervouet et al., 2005, Zhang et al., 2007), and confers the functional advantage of suppressing respiration in an oxygen-poor environment. vHL/FIH KO MEFs (Figure 4G) differ from both single FIH and vHL KOs, with a lower basal respiration rate, as well as a reduced spare respiratory capacity. The loss of both of these negative regulators of HIF response has the capacity to switch cells almost fully to a highly glycolytic state, yet interestingly, double deletion also appears to allow a greater sparing of respiratory capacity.

The effect of FIH loss on respiration is essentially eliminated by culture at 1% oxygen for 72 hr (Figure 4H). This indicates that the increased basal respiration and increased respiratory capacity are suppressed by hypoxic response; interestingly, the respiratory response seen in these cells is essentially identical to that seen in FIH/vHL null cells under normoxic conditions in Figure 4G. This would argue that the chief modulators of metabolic change in respiration under hypoxia are these two negative regulators of the HIF pathway. As seen in Figures 4B and 4D, the glycolytic shift is more complex in this regard, although another interpretation of this would be that a full equivalence between glycolytic rate and glycolytic reserve would require an even lower degree of hypoxia than 1% oxygen conditions provide.

The increased oxygen consumption in FIH null MEFs is not due to an increased capacity for fatty acid oxidation (Figure 4I), as FIH null cells in fact have lower basal respiration and lower oxidative reserve capacity than control cells when supplied solely with palmitate as a substrate for oxidation. This experiment indicates that FIH null cells are unable to fully switch to fatty acid oxidation. In addition, when both glucose and palmitate are absent, FIH null cells have the same spare respiratory capacity as WT cells, but a lower basal oxygen consumption. This means that WT cells are oxidizing a substrate in the basal state that FIH null cells cannot, a substrate that cannot be used by the mutant cells to increase spare respiratory capacity.

These data indicate that FIH null cells have a block in fatty acid oxidation and likely oxidation of other substrates. This, coupled to the data in Figure 4A, argues that FIH null cells channel as much glucose as possible through pyruvate to the mitochondria due to this block, and thus have a lower rate of lactate production as a result at baseline than, for example, vHL null cells. However, when respiration is blocked by oligomycin, the increase in lactate production reveals the higher rate of glycolysis in these cells. Despite a low capacity for fatty acid oxidation, the finding that FIH null cells maintain higher ATP levels with preserved ATP:AMP ratios suggests that the alternate oxidative pathways FIH null cells channel their large glycolytic reserve into, and use to make up for their diminished fatty acid oxidation, are energetically favorable.

### Loss of FIH in Skeletal Muscle Demonstrates Its Role in the Potentiation of Hypoxic Metabolic Shifts

**Figure 5:**
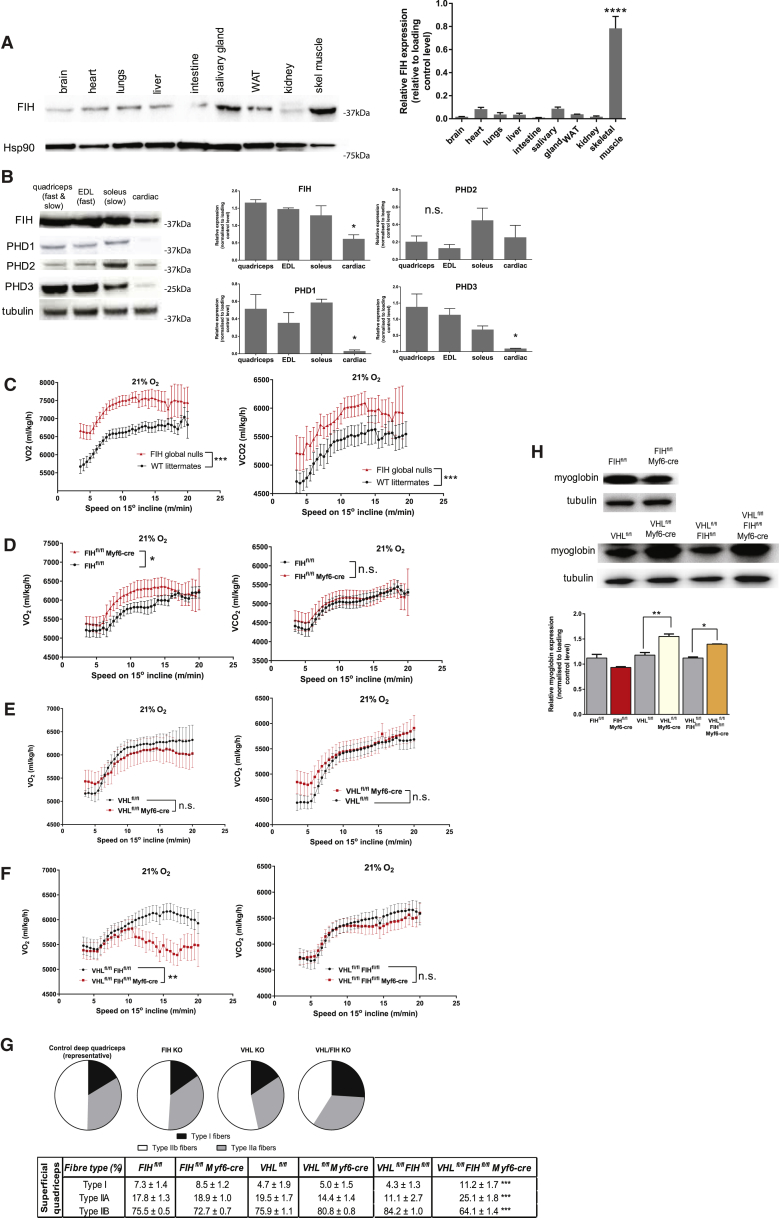
*In Vivo* Effects of FIH Loss in Mouse Skeletal Muscle Mirror Its Metabolic Roles in Cells (A) Representative immunoblot of FIH in whole organ lysates in a WT mouse, demonstrating different levels of FIH in different tissues. (B) Representative immunoblot of molecular oxygen sensors in whole muscle lysates taken from a WT mouse. Various hindlimb muscle samples were taken from male WT mice: the predominantly fast twitch extensor digitorum longus (EDL), predominantly slow twitch soleus, and quadriceps comprising both fast and slow twitch fibers. Cardiac muscle was sampled separately. (C) FIH nullizygous mice show increased oxygen consumption (VO_2_) and carbon dioxide production (VCO_2_) rates on an incremental uphill treadmill protocol. (D) VO_2_ and VCO_2_ measured during an incremental uphill exercise protocol in FIH skeletal muscle mutant versus control littermate mice over a range of exercise intensities, suggesting oxygen inefficiency. In contrast, the VCO_2_ curve was not significantly altered. A two-tailed Student's t test was applied to equations of curves fitted separately to control and mutant mice measurements. (E) vHL skeletal muscle mutant mice did not show the qualitative VO_2_ differences observed in FIH muscle-specific mutants. (F) vHL/FIH muscle double-KO mice show a fall-off in VO_2_ measurements at low exercise intensities. In contrast, their VCO_2_ is not altered. (G) Fiber type composition in cross-sections of frozen mouse superficial quadriceps from each genotype. Concurrent loss of FIH and vHL shifts fiber identity in deep quadriceps toward slow twitch, more oxidative fiber types. Six frozen sections were analyzed per mouse. (H) The loss of vHL leads to an increase in myoglobin expression in whole-quadriceps lysates of vHL KO muscle and vHL/FIH double KO, while the loss of FIH has no discernible effect. ^∗^p < 0.05, ^∗∗^p < 0.01, ^∗∗∗^p < 0.001, ^∗∗∗∗^p < 0.0001; n.s., not significant. Data are represented as means ± SEM. Only male mice were used for *in vivo* experiments, with male littermates as controls. n = 7 mice/genotype, unless otherwise stated. For multiple comparisons (A, B, G, and H), a one-way ANOVA was used. For pairwise comparisons (C–F), a two-tailed Student's t test was used. After curve-fitting, best-fit equations were compared with an extra sum-of-squares F test. See also Figure S4.

Based on the data above, we postulated that tissues experiencing rapid and dynamic fluxes in oxygenation, and that need to rapidly adjust their metabolic activity in response to those fluxes, would be those most sensitive to the loss of FIH function. Indeed, there is significant variation in FIH expression across tissues of the mouse, with by far the highest levels of expression found in skeletal muscle (Figure 5A). These elevated levels are restricted to skeletal muscle, not being seen in intestinal smooth muscle or cardiac muscle.

Interestingly, FIH expression is uniformly high in both fast and slow twitch muscle, whereas among the PHD isoforms, PHD3 levels are highest in predominantly fast twitch muscles, and PHD2 in the soleus, a predominantly oxidative, slow twitch muscle (Figure 5B). These data support HIF-1α’s role in regulating skeletal muscle metabolism (Mason et al., 2004, Mason et al., 2007). To determine the relationships between FIH and metabolic function *in vivo*, we carried out studies of treadmill regulated running on mice deficient in FIH/vHL.

To understand basal roles of FIH in exertion-induced metabolic shifts, we first analyzed FIH global deletion animals. As previously shown, these animals had a higher basal metabolic rate (Zhang et al., 2010), seen here during the initiation of exertion (Figure 5C). This elevated metabolic rate is also evident over increasing speeds in an uphill treadmill protocol (Figure 5C). Global deletion of vHL is not compatible with postnatal viability, and so could not be assayed in the same manner.

We then generated mice with skeletal muscle-specific deletions of FIH, vHL, or both, via employment of an Myf6-promoter-driven cre recombinase transgene (Haldar et al., 2008, Keller et al., 2004) (Figure S4A). The use of this cre recombinase was necessary because deletion of vHL with the striated muscle-specific myosin creatine kinase (CKMM) cre recombinase leads to mid-gestational lethality in vHL muscle-specific mutants (data not shown), likely because vHL loss from cardiac muscle (cardiac deletion occurs to a certain extent in the CKMM cre strain) has deleterious effects (Lei et al., 2008). Analysis of singular FIH and vHL mutants showed no specific constitutive shifts in basal metabolic rate, although FIH skeletal muscle mutants had a higher nocturnal VO_2_, when activity of mice is highest, and vHL mutants tended toward higher VCO_2_ nocturnally (Figures S4B–S4E).

During exercise, loss of FIH specifically in skeletal muscle creates an increase in VO_2_, analogous to the effects of global FIH loss (Figure 5D). Loss of skeletal muscle vHL, conversely, causes a decreased VO_2_ (Figure 5E), and loss of FIH/vHL causes a sharp fall-off in VO_2_ at higher running speeds (Figure 5F). Conversely, there is a steeper rise in respiratory exchange ratio (RER) with running speed in vHL and FIH/vHL KO groups, suggesting that they are more acutely dependent on carbohydrate conversion, as opposed to fatty acid oxidation, for power output (Figure S4F). These data draw analogies between metabolic alterations described above in MEFs and resultant effects on whole-animal metabolism during exercise in tissue-specific mutants: that loss of FIH increases oxygen uptake, loss of vHL suppresses it, and the loss of both causes an accelerated response that dramatically reduces oxidative metabolism.

There are anatomic effects of the loss of both vHL and FIH in skeletal muscle: while the loss of each singly has no discernable effect on fiber type, the loss of both shifts fiber type identity in superficial quadriceps away from type IIb, or fast twitch, and toward type I and type IIa, and thus more oxidative fiber types (Figure 5G). This shift is less marked in skeletal muscle, which is already mainly oxidative, e.g., deep quadriceps (Figure S4G). Furthermore, vHL loss increases myoglobin expression in skeletal muscle, regardless of its FIH status (Figure 5H). Both these changes are in contrast to the role of FIH/vHL loss in suppressing oxidative metabolism, and may be compensatory.

### FIH Loss Accelerates Hypoxic Adaptation in Skeletal Muscle

**Figure 6:**
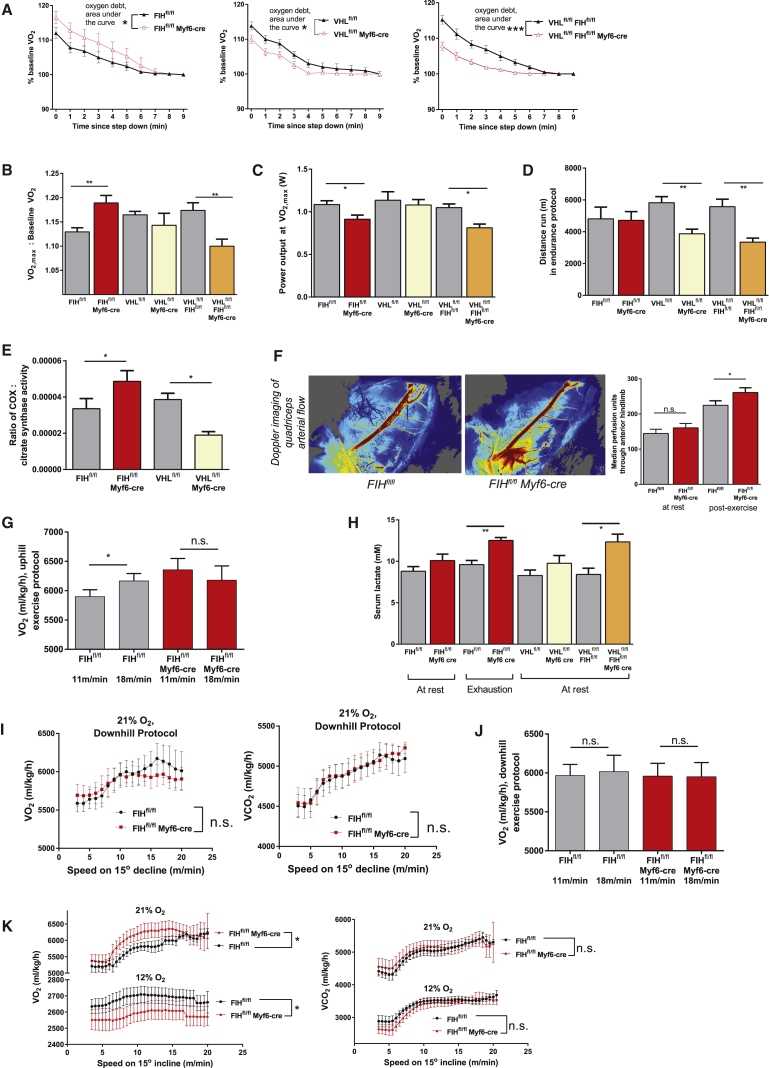
Effects of FIH Loss on Specific Oxidative Parameters in Male Mouse Skeletal Muscle (A) Oxygen debt in male mice of genotypes indicated, following a running protocol of fixed length. n = 5 mice per genotype. After curve-fitting, best-fit equations were compared with an extra sum-of-squares F test. (B) Normalized VO_2,max_ values in male mice of genotypes indicated, following an incremental uphill running protocol. (C) FIH muscle mutant male mice and vHL/FIH double-KO male mice achieve VO_2,max_ at lower exercise intensities compared with controls. (D) Endurance performance on a distance running protocol. (E) Cytochrome oxidase (COX) activity in gastrocnemius muscle lysates from male mice of various genotypes. (F) Local quadriceps perfusion measured by Doppler imaging before and after an uphill running protocol where male mice were run to exhaustion. (G) Changes in VO_2_ with running intensity within an uphill running protocol. (H) Relationship between blood lactate levels in resting mice and genotype. Blood lactate levels rose more significantly in FIH muscle-specific mutant male mice, following an incremental uphill treadmill protocol where mice were run to exhaustion. (I) Downhill running protocol. Relationships between VO_2_ and VCO_2_ measurements and downhill running speed. After curve-fitting, best-fit equations were compared with an extra sum-of-squares F test. (J) Changes in VO_2_ with running intensity within a downhill running protocol. (K) Relationship between running speed and VO_2_ or VCO_2_, when running under 12% oxygen versus under room air. A two-tailed Student's t test was applied to equations of curves fitted separately to control and mutant mice. After curve-fitting, best-fit equations were compared with an extra sum-of-squares F test. ^∗^p < 0.05, ^∗∗^p < 0.01, ^∗∗∗^p < 0.001; n.s., not significant. Data are represented as means ± SEM. Only male mice were used for *in vivo* experiments, with male littermates as controls. n = 7 mice/genotype, unless otherwise stated. For multiple comparisons (B–E), a one-way ANOVA was used. For pairwise comparisons (A, I, and K), a two-tailed Student's t test was used. For grouped data (F–H and J), a two-way ANOVA was used. See also Figure S5.

FIH skeletal muscle deletion mutants have an increased oxygen debt post-exercise, whereas both vHL and FIH/vHL mutants have a decreased debt (Figure 6A). This again is correlated with the increased oxidative metabolism of FIH skeletal muscle null mutants.

The loss of FIH gives rise to an increase in VO_2max_ relative to the baseline VO_2_, whereas this trends toward a decrease in vHL skeletal muscle mutant animals and a significant decrease in FIH/vHL mutants (Figure 6B). In these analyses, loss of FIH is correlated with FIH mutant mice achieving VO_2max_ at a lower speed than control mice do, and although this was not seen in vHL mutants, it was seen in FIH/vHL null mutants (Figure 6C). This may allow FIH null muscle to conserve glycogen stores at lower exercise intensities, in favor of deploying them at higher exercise intensities, where anaerobic metabolism would be more useful. Interestingly, while loss of muscle FIH causes a decreased oxygen efficiency at lower exercise intensities, it does not reduce endurance in a long-distance running protocol (Figure 6D). This is in contrast with loss of vHL and FIH/vHL, which both reduce endurance significantly relative to control littermates.

Two improvements in hypoxic adaptation could serve to maintain exercise performance in the FIH skeletal muscle mutant, despite the decreased efficiency observed. There is a specific increase in relative COX activity ratios in FIH mutant muscle extracts (Figure 6E). Furthermore, Doppler imaging of muscle perfusion (Figure 6F) revealed that, following exercise to exhaustion, loss of FIH causes an increase in overall perfusion of the exposed muscle compared with littermate control animals—part of a potentiated response to the metabolic stress of exercise.

Comparing exercise at moderately low and moderately high exercise intensities, while the loss of FIH leads to a high VO_2_ at 11 m/min, this VO_2_ is more quickly suppressed in mutants as exercise intensity is increased to 18 m/min (Figure 6G). This suggests that the loss of FIH creates first an increase in VO_2_ during exercise, but subsequently acts to suppress oxygen uptake, allowing anaerobic metabolism at higher exercise intensities. This acceleration of metabolic response is also evident in the finding that mice lacking skeletal muscle FIH have a significantly greater increase in serum lactate levels following exercise to exhaustion (Figure 6H). This is correlated with a greater increase in serum creatine kinase in these mice, indicative of increased anaerobic stress (Figure S5A). Interestingly, there is a greater shift in the lipid metabolome in FIH mutant muscle than in the overall metabolome post-exercise (Figure S5B), suggesting that differential effects on lipid metabolism likely underlie the metabolic shifts seen in the mutants, and are potentially facilitating the oxidative shifts seen.

As eccentric exercise utilizes glycolytic muscles more intensively (Nardone and Schieppati, 1988), we compared the performance of FIH muscle mutant and control mice on downhill treadmill running. VO_2_ in FIH mutants was more blunted than VCO_2_ in comparison with controls (Figure 6I). This differed significantly from that seen in uphill (concentric) running. Consistent with FIH's role in accelerating hypoxic adaptation, RER of FIH KO animals had a steeper relationship with running speed in downhill exercise (Figure S5C) than in the uphill exercise protocol described above.

Further support for a model wherein FIH loss accelerates metabolic adaptation was seen from studies in which mice exercised in 12% oxygen. While the loss of FIH increases VO_2_ under normoxia, under inhalational hypoxia this is suppressed, with a *decrease* in VO_2_ during exercise (Figure 6K). This result demonstrates that the loss of FIH accelerates the inhibition of oxidative metabolism seen in hypoxic muscle tissue. This is analogous to the suppression of spare respiratory capacity shown in Figure 4, where FIH null cells exposed to prolonged hypoxia consume as little oxygen as hypoxic control cells. The mechanism of this is elusive; given its oxygen sensitivity it likely encompasses changes in mitochondrial gene expression (Figure S5D), but it is unrelated to gross mitochondrial structure (Figure S5E).

## Discussion

Metabolic shifts in response to changes in energy demand are complicated by oxygen availability within tissues. HIF transcription factors modulate this relationship between metabolic response and oxygen partial pressures. There are two key post-translational mechanisms that regulate HIF activity in a directly oxygen-dependent manner; we show here that these pathways act synergistically to modulate metabolism.

Previous studies suggested that FIH impairment upregulates glycolytic genes (Sakamoto et al., 2011, Sakamoto et al., 2014, Wang et al., 2014), although not in normoxia (Bracken et al., 2006). In line with this, we show that cells lacking FIH have a greater capacity than control cells to increase glycolysis when oxidative metabolism is compromised. We also show that FIH null cells do not have the same capacity as WT cells for fatty acid oxidation, indicating that increased glycolysis is likely channeled more exclusively to pyruvate and not to lactate in these cells as part of a compensatory metabolic shift. The increase in respiratory capacity caused by FIH loss is more surprising, given the well-documented suppression of aerobic metabolism and performance with vHL/PHD loss (Fukuda et al., 2007, Kim et al., 2006). This indicates that FIH plays a fundamentally different role in regulating metabolic responses than that played by the PHD/vHL axis of HIF control.

Our cells lacking FIH show no significant increase in glucose uptake, despite an increase in glycolytic reserve that almost matches that seen in cells lacking vHL. There is strong evidence to show that oxidative activity increases when certain steps of glycolysis are inhibited selectively, e.g., immediately after hexokinase is inhibited with 2-deoxyglucose (Wu et al., 2006). Other potential mechanisms for increased oxidative metabolism include shifts in metabolic gene expression. This could conceivably happen through a threshold-dependent aspect of HIF-driven gene expression, first toward, and then away from, oxidative metabolism, or through novel substrates that straddle metabolic pathways (Cockman et al., 2006, Scholz et al., 2015, Wilkins et al., 2012, Yang et al., 2013).

Our data indicate that FIH loss accelerates specific metabolic adaptations to hypoxia. We showed that animals without skeletal muscle FIH have lower rates of oxidative metabolism when exercising in environmental hypoxia, and when under the glycolytic stress of downhill running. In these animals, a loss of FIH has clearly exaggerated a suppression of oxidative metabolism. This leads to a unifying model for FIH function: loss of FIH activity speeds up oxidative processes; this reduces intracellular levels of oxygen, acting to potentiate PHD inhibition, and thus HIF-1α accumulation. An accelerated hypoxic metabolic adaptation then, ultimately, suppresses oxidative metabolism via HIF accumulation. As the FIH enzyme is thought to have a higher oxygen affinity than the PHD enzymes, ordinarily the loss of PHD activity and loss of FIH activity should occur sequentially, and in the wrong order to support this model. However, the differential affinities of the PHD enzymes and FIH might be less relevant where oxygen levels drop suddenly, a situation in which HIF levels need to be elevated as rapidly as possible. In such a case, whatever remaining oxygen there is in the cell would need to be removed for the cell to remain oxidative as long as possible, and then to as rapidly as possible accumulate HIF and effect a shift to glycolysis. It could as well be possible that FIH inhibition uses mitochondria as oxygen sinks, to propagate the cycle of oxygen depletion and a subsequent PHD/HIF response spatially, e.g., to the muscle cell surface, where some oxygen reserves remain on myoglobin even under significant hypoxia (Takahashi and Asano, 2002).

This model would predict that tissues with the greatest need to retard hypoxic response as long as possible, but then activate it as rapidly as possible, would also be those that express the most FIH. As we have shown, skeletal muscle has by far the highest levels of FIH protein found in the body. Considered alongside high HIF levels in fast twitch fibers (Pisani and Dechesne, 2005), and lower HIF levels in slow twitch fibers, successful clamping of HIF activity could also underlie differential HIF activation in muscle metabolism. Consistent with this, our group has shown that FIH expression is significantly higher in athletes undergoing endurance training (Lindholm and Rundqvist, 2016, Lindholm et al., 2014). Further work in this area may show relevance to other aspects of human physiology, e.g., certain FIH polymorphisms being enriched in populations at altitude (Ji et al., 2012). It is also interesting to note that the exercise intolerance noted in Chuvash polycythemia patients (Formenti et al., 2010) is analogous to those seen in skeletal muscle vHL deletion mice.

A corollary of our findings and this model would be that, under conditions of rapid reoxygenation, a rapid suppression of FIH activity could be critical in re-establishing oxidative metabolism in the presence of accumulated HIF. An acceleration of oxygen consumption could itself temper the consequences of reoxygenation, e.g., by maintaining a basal, protective level of HIF activity. Future experiments will address this; one could imagine this would be key in understanding metabolic adaptations during ischemia and reperfusion.

### Limitations of Study

In this study, we have not delineated catalytic versus non-catalytic actions of FIH; future studies will endeavor to determine how the hydroxylase acts in an oxygen-dependent manner to induce the metabolic roles of FIH, and the extent to which, for example, protein-protein interactions via the JmjC domain are responsible. In addition, the role of such interaction in sequestering FIH may be key in understanding how its hydroxylase activity could be restricted within cells.

A key concern here is the overall role of the hydroxylation of HIF, and thus the direct role played by oxygen. Functions of FIH outside of its activity as a hydroxylase will need to be fully explored, as these will illustrate the degree to which it is acting in an oxygen- and HIF-dependent manner. In the context of hydroxylation, there is some disagreement as to the actual oxygen affinity of the FIH enzyme in *in vivo*, and, here, defining the spatial and sub-cellular distribution of FIH will be critical, particularly if that differs substantially from tissue to tissue. Finally, non-HIF targets of FIH have been suggested by a number of researchers, and thus a final definition of the mechanisms of FIH action on oxidative metabolism will require a detailed inclusion or exclusion of putative FIH targets with a potential metabolic function.

## STAR★Methods

### Key Resources Table

**Table:** 

REAGENT or RESOURCE	SOURCE	IDENTIFIER
**Antibodies**		

Rabbit polyclonal anti-alpha tubulin, 1:2000	Cell Signalling	Cat#2144; RRID: AB_1968816
Mouse monoclonal anti-beta actin, 1:2000	Sigma	Cat#A5316; RRID: AB_476743
Rabbit polyclonal, anti-FIH, 1:1000	Abcam	Cat#ab36814; RRID: AB_869843
Rabbit polyclonal, anti-Hsp90, 1:1000	Cell Signalling	Cat#4874; RRID: AB_2121214
Rabbit polyclonal, anti-mTOR, 1:1000	Cell Signalling	Cat#2983; RRID: AB_2105622
Rabbit polyclonal anti-PGC1a, 1:1000	Santa Cruz Biotechnology	Cat#sc-13067; RRID: AB_2166218
Rabbit polyclonal anti-PHD1 1:1000	Bethyl Laboratories	Cat#A300-326; RRID: AB_2096867
Rabbit polyclonal anti-PHD2, 1:1000	Novus Biologicals	Cat#NB100-2219; RRID: AB_578125
Rabbit polyclonal anti-PHD3, 1:1000	Novus Biologicals	Cat#NB100-303; RRID: AB_350220
Mouse monoclonal anti-VHL, 1:500	BD Pharmingen	Cat#556347; RRID: AB_396376
Anti-mouse IgG-HRP Donkey, 1:5000	Santa Cruz Biotechnology	Cat#sc-2314; RRID: AB_641170
Anti-rabbit IgG-HRP Donkey, 1:5000	R&D Systems/ ThermoScientific	Cat#SA1-200; RRID: AB_325994
Goat polyclonal anti-FIH (overexpression experiments), 1:200	Santa Cruz Biotechnology	Cat#sc-26219; RRID: AB_2117262
Goat polyclonal anti-FIH (for mitochondrial complex profiling), 1:500	Santa Cruz Biotechnology	Cat#sc-26219; RRID: AB_2117262
Mouse monoclonal anti-paxillin (5H11) (overexpression experiments), 1:1000	Upstate Biotechnology/Millipore	Cat#05-417; RRID: AB_309724
Mouse monoclonal anti-lamin B1 (B-10), 1:500	Santa Cruz Biotechnology	Cat#sc-374015; RRID: AB_10947408

**Bacterial and Virus Strains**		

Ad-GFP	Vector Biolabs	Cat#1060
Ad-Cre-GFP	Vector Biolabs	Cat#1700

**Chemicals, Peptides, and Recombinant Proteins**		

7’-aminoactinomycin D	eBioScience	Cat#00-6993-50
MitoSOX	Thermo Fisher Scientific	Cat#M36008
Tetramethylrhodamine methyl ester	Thermo Fisher Scientific	Cat#T668
RNase A	Qiagen	Cat#19101
Perchloric acid, 60%	Sigma	Cat#311413
Potassium phosphate monobasic	Fisher	Cat#P2853
Potassium phosphate dibasic	Fisher	Cat#P290500
Tetrabutylammonium-bisulfate	Sigma	Cat#T7158
HPLC-grade chromatography water	Fisher	Cat#AC268300025
L-carnitine	Roche	Cat#11242008001
Sodium palmitate	Sigma	Cat#P9767
(+)-Etomoxir sodium salt hydrate	Sigma	Cat#E1905
Seahorse XF modified DMEM media	Agilent Technologies	Cat#102365-100
Seahorse XF assay calibrant	Agilent Technologies	Cat#100840-000
Calcein AM dye	Life Technologies	Cat#C3100MP
Amersham ECL Western Blotting Detection Reagent	GE Healthcare	Cat#GERPN2124
CelLytic M (previously Cell Lysis M Reagent)	Sigma	Cat#C2978
Nitro-blue tetrazolium	Sigma	Cat#11585029001

**Critical Commercial Assays**		

Glucose oxidase assay	Merck/Calbiochem,	Cat#CBA086
Lactate dehydrogenase assay	Cayman Chemical	Cat#600450
Citrate synthase activity assay	Sigma	Cat#CS0720
Cytochrome c oxidase activity assay	BioVision	Cat#K287-100
XF Mito Stress Test Kit	Agilent Technologies	Cat#103015-100
XF Glycolytic Stress Test Kit	Agilent Technologies	Cat#103020-100
Total OXPHOS Rodent WB Antibody Cocktail	Abcam	Cat#110413

**Deposited Data**		

Affymetrix microarray data, FIH, VHL and VHL/FIH null mouse embryonic fibroblasts	Zhang et al. (2010)	GEO: GSE20335

**Experimental Models: Organisms/Strains**		

Mouse: VHL fl/fl: C;129S-Vhl<tm1Jae>/J	The Jackson Laboratory	004081
Mouse: MCK cre: B6.FVB(129S4)-Tg(Ckmm-cre)5Khn/J	The Jackson Laboratory	006475
Mouse: Myf6 cre: B6;129-Myf6<tm2(cre)Mrc>/J	The Jackson Laboratory	010528
Mouse: FIH nullizygous	Zhang et al. (2010)	N/A
Mouse: FIH fl/fl	Zhang et al. (2010)	N/A

**Oligonucleotides**		

See Table S1	N/A	N/A

**Recombinant DNA**		

gBlock double-stranded DNA containing mouse FIH	Integrated DNA Technologies	N/A
Plasmid: pEF_IRES_puro6	Department of Biochemistry, University of Adelaide	N/A

**Software and Algorithms**		

NMR Suite 7.6	Chenomx	http://www.chenomx.com/
GenePattern Suite	The Broad Institute	http://software.broadinstitute.org/
Fusion FX System (blot acquisition)	Vilber Lourmat	https://www.vilber.com/
FlowJo vX (flow cytometry acquisition)	FlowJo, LLC	https://www.flowjo.com/
XF Hypoxia Rate Calculator Program	Agilent Technologies	http://www.agilent.com/
FLPI, Laser Speckle Contrast Imager	Moor Instruments	http://moor.co.uk/
ImageJ	NIH	https://imagej.nih.gov/ij/
Waters Q-ToF Xevo acquisition software	Waters Corporation	http://www.waters.com/
Ultra Performance Liquid Chromatogram acquisition software	Waters Corporation	http://www.waters.com/

**Others**		

SCI-tive Hypoxia Workstation	Baker Ruskinn	N/A
XF24-3 Analyzer	Agilent Technologies	N/A
Oxymax indirect calorimetry system with modular treadmill setup	Columbus Instruments	N/A

### Contact for Reagent and Resource Sharing

Further information and requests for resources and reagents should be directed to the Lead Contact, Randall S. Johnson (rsj33@cam.ac.uk).

### Experimental Model and Subject Details

#### Mice

Animal work was carried out under UK Home Office guidelines. Mice were housed in a pathogen-free animal facility, provided with food and water *ad libitum*, (normal maintenance mouse chow from: SAFE Diets, catalog #A04), and maintained on a 12h light-dark photoperiod at a regulated 21 degrees C. Mice were genotyped with DNA from ear biopsies, using either in-house PCR or commercial Transnetyx qPCR assays. Mice carrying the FIH gene where exon 2 is flanked by loxP sites (FIH^fl/fl^) were described previously (Zhang et al., 2010). Mice possessing loxP sites flanking the VHL promoter and exon 1 (VHL^fl/fl^) were acquired from The Jackson Laboratory’s repository (JAX 004081). All mice were backcrossed into the C57/BL6J background over at least four generations. Breeding pairs where both breeders were homozygous for either the floxed FIH (FIH^fl/fl^) or the floxed VHL allele (VHL^fl/fl^) were used to generate FIH^fl/fl^ and VHL^fl/fl^ mice/embryos. FIH^fl/fl^ and VHL^fl/fl^ mice were crossed over three generations to derive FIH^fl/fl^VHL^fl/fl^ mice/embryos.

FIH^fl/fl^ or VHL^fl/fl^ mice were crossed with transgenic mice expressing cre recombinase under the control of either the striated muscle-specific creatine kinase CKMM promoter (JAX 006475) or the skeletal muscle-specific Myf6 promoter (JAX 010528), which were also acquired from The Jackson Laboratory and 010528 respectively). As VHL^fl/fl^-CKMM cre mice did not survive to birth, VHL^fl/fl^-Myf6 cre were likewise generated. Within three generations, skeletal muscle-specific FIH null (FIH^fl/fl cre^) or VHL null (VHL^fl/fl^) mice were derived and used to set up experimental crosses. Only 3 month-old male mice were used *in vivo* exercise assessments. Age-matched male littermates (FIH^fl/fl^, VHL^fl/fl^) were used as controls. FIH^fl/fl^VHL^fl/fl^ animals were generated in the C57/BL6J background by crossing FIH^fl/fl^ and VHL^fl/fl^ animals, and subsequently offspring across 2-3 generations carrying both floxed alleles. After crossing the respective cre recombinase transgenes into the FIH^fl/fl^VHL^fl/fl^ lineage, experimental crosses were set up as follows: FIH^fl/fl^VHL^fl/fl^ X FIH^fl/fl^VHL^fl/fl^ cre.

FIH^Δ2/+^ mice described previously by our group (Zhang et al., 2010) were reconstituted from cryopreserved sperm, and backcrossed for four generations to the C57/BL6J background. FIH^Δ2/+^ heterozygous experimental crosses were used to derive sex-matched FIH nullizygous (FIH^Δ2/Δ2^) males with male wildtype littermates as controls.

#### Derivation of Primary Mouse Cells

Primary mouse embryonic fibroblasts (MEFs) were isolated from macerated E12.5-13.5 embryos of the relevant genotypes (whole litter of embryos used)(mixed cultures were created without selection for gender) and immortalized by stable transfection with SV40 large T antigen at passage 3. Cells were then subcultured over seventeen more passages. Unless otherwise stated, MEFs were cultured in a humidified 37°C atmosphere of 21% oxygen, 5% carbon dioxide, in high glucose DMEM (Invitrogen) supplemented with 10% fetal bovine serum (Gibco), penicillin and streptomycin.

#### Generation of an FIH Knockout MEF Cell Line Stably Overexpressing Mouse FIH

A gBlock double-stranded DNA containing mouse FIH with 40 bp complementary ends (Integrated DNA Technologies) was cloned via Gibson assembly into a pEF_IRES_puro6 expression vector. FIH^-/-^ MEFs cultured without penicillin and streptomycin in 100 mm dishes were transfected at 90% confluency with 30 μg of either a pEF_IRES_FIH_puro6 or an empty vector. Two days after transfection, cells were selected with 2 μg/ml puromycin for two weeks prior to analysis by western blotting and use in Seahorse assays.

### Method Details

#### Acute Deletion of Target Genes from MEFs

1x10^6^ immortalized VHL^fl/fl^ FIH^fl/fl^ MEFs were plated briefly, then infected overnight with 100 pfu/cell of adenovirus expressing under the control of a CMV promoter either eGFP alone (Vector Biolabs), or both eGFP and cre recombinase (Vector Biolabs). After washing in DPBS, infected cells were trypsinized. The cell population was enriched for eGFP-positive cells using a MoFlo cell sorter (DakoCytomation). For this study, knockouts were only compared to control cell populations derived from the same parent ‘floxed’ cell population. One passage post-infection, total genomic DNA was purified from a sample of cultured cells using a DNeasy kit (Qiagen) with RNaseA treatment. Deletion efficiency was quantified with qPCR using TaqMan (Roche) reagents, comparing FIH and VHL to HIF1α gDNA levels in wildtype and knockout cells. qPCR analysis was performed with an ABI StepOne Plus detection system.

#### *In Vitro* Hypoxic Treatment of MEFs

For hypoxic studies, cells were seeded and cultured in normoxia (21% oxygen) until they were transferred to a temperature-, humidity- and gas-controlled workstation (Baker Ruskinn) with an atmosphere of 5% CO_2_ and 1% O_2_. To permit comparison across timepoints in a single hypoxic timecourse, all cell samples were cultured for the same total length of time, and transferred into hypoxia only for the designated timeframe at the end of the protocol. To avoid reoxygenation, RNA, protein, metabolite and media samples were collected within the hypoxic workstation.

#### ^1^H-NMR Analysis of Aqueous Metabolites

Aqueous metabolite concentrations were measured in 600μl culture media aliquots. To extract aqueous metabolites from cell lysates, cell culture media was aspirated and adherent cells washed twice with ice-cold DPBS. Cell layers were immediately scraped into cold 6% perchloric acid (Sigma), then neutralized completely with 10M KOH. The supernatant was lyophilized and stored until analysis, where they were reconstituted in 600μl D_2_O. Metabolite concentrations in both media and cell lysates were eventually normalized to live cell counts in duplicate wells plated under the same original conditions.

^1^H-NMR spectroscopy was performed on a 600 MHz Bruker Avance NMR spectrometer. Solvent suppression pulse sequence was used for acquisition of the ^1^H-NMR data. DSS was used as an internal standard. Spectral processing consisted zero- and first-order phase corrections followed by baseline correction using NMR Suite 7.6 (Chenomx). Metabolites were identified by chemical shift assignments using the same interface. The absolute concentration of metabolites was calculated by normalizing peak area to the concentration of DSS in each sample calculated by an ERETIC method.

#### Microarray Data Interpretation

An existing Affymetrix microarray dataset relevant to FIH KO, VHL KO and VHL/FIH dKO mouse embryonic fibroblasts was downloaded from NCBI GEO (GEO: GDS3769). A principal component analysis was first performed using the relevant function on GenePattern (Broad Institute, USA) server, to visualize differences. The dataset was filtered through a PreprocessDataset function on GenePattern, applying lower and upper limits of 0 and 20 000 to Affymetrix results. Genes with minimal variability across samples (absolute fold change of less than 2 or a range of less than 50) were excluded from analysis. Using GenePattern’s ComparativeMarkerSelection and ExtractComparativeMarkerResults modules, pairs of genotypes were subject to a two-sided t-test without permutation.

Taking each knockout / control pair in turn, the fold change and p-value of each gene were plotted against each other using the MultiPlot module on GenePattern. To extract metabolically relevant information from this dataset, genes with KEGG metabolic pathway gene ontologies were identified on data plots for clearer visualization.

#### RNA Extraction from Cells and qRT-PCR Analysis

Total RNA was isolated from cultured cells using an RNeasy kit (Qiagen), with accompanying DNase I treatment (Qiagen). 1μg RNA was reverse-transcribed using a SuperScript III First Strand Synthesis system (Invitrogen). Resulting cDNA was then diluted 1:20, and amplified with qPCR using SYBR Green (Roche) reagents in an ABI StepOne Plus detection system. Cycling conditions: Heat ramp 95°C x 10min, extension (95°C x 15s, 60°C x 1min) x 40 cycles, melt curve 95°C x 15s, 60°C x 1min, 95°C x 15s with 0.3°C increments. Primer sequences are appended (Table S1). Fold change gene expression was calculated by normalisation to 18S, i.e. 2^-ΔΔCT^ = 2^-[(CT,target – CT,housekeeping)hypoxia - (CT,target – CT,housekeeping)normoxia]^.

#### Apoptosis Assay

MEFs (150 000) were seeded per well of a 6 well plate. To measure apoptosis, MEFs were exposed to various levels of hypoxia, trypsinized at various timepoints, incubated with 7’-AAD (1:20) (eBioScience) in DPBS at 4°C for 15min, then analyzed for the percentage of dead (positively-stained) cells using flow cytometry.

#### Enzyme-Based Metabolic Assays

MEFs (150 000) were cultured for up to 72h in 21% or 1% oxygen. A media sample was taken at different timepoints for colorimetric quantification of glucose concentration by a glucose oxidase assay (Merck/Calbiochem), and lactate concentration by a lactate dehydrogenase assay (Cayman Chemical), according to manufacturers’ instructions. Glucose and lactate measurements from unconditioned media were used for comparison. Adherent MEFs were washed and lysed in a non-denaturing Cell M Lysis Reagent (Sigma) containing proteinase inhibitor. Lysates were centrifuged, and the supernatant kept at -80°C until analysis. Native citrate synthase activity (Sigma) and cytochrome c oxidase activity (BioVision) in lysates was measured using colorimetric kinetic measurements according to manufacturers’ instructions, using a Tecan Sunrise microplate-reader. In particular, COX activity was measured by following the oxidation of reduced cytochrome c as an absorbance decrease at 550nm. All readings were then normalized to viable cell counts obtained from duplicate wells plated under the original conditions.

#### Flow Cytometric Analysis

To characterize various mitochondrial parameters in MEF populations, MEFs were cultured for 48h, then trypsinized and stained with one of the following at 4°C over 20min in DPBS: 10μM MitoSOX (Thermo Fisher), 200nM tetramethylrhodamine methyl ester (Thermo Fisher). Stained cells were then washed and kept in cold DPBS briefly before analysis. Results were acquired with a Fortessa (Becton-Dickinson) flow cytometer, and analyzed in FlowJo vX.

#### Quantification of mtDNA

Total genomic DNA was extracted from cultured cells using a DNeasy kit (Qiagen) with RNaseA treatment. To approximate mtDNA content (Huo and Scarpulla, 2001, Shen et al., 2004), the relative quantities of mtDNA genes encoding cytochrome B and mCO-1, and rDNA genes encoding 18S and m5S, were determined by qPCR. A list of primer sequences is appended (Table S1).

#### HPLC Assay for High Energy Nucleotides

Cell culture media was removed and cells were washed twice with ice-cold DPBS. Cell layers were scraped into ice-cold 6% perchloric acid. A known amount of cytidine monophosphate (Sigma) was added as an internal standard at the same time. Cells were then immediately scraped and collected. Samples were incubated on ice for 10min, then centrifuged. Universal indicator solution (Fisher) was added to supernatants. The samples were neutralized with KOH and the supernatants were collected and lyophilized.

For analysis, each sample was reconstituted in 200-300μl of HPLC-grade water (Fisher), then 20μl was injected onto a 3.0 μm SUPELCOSIL LC-18-T HPLC column (Sigma) using a Dionex Ultimate 3000 system. The column temperature was held at 30°C and the flow rate was 1.0 ml/min. Buffer A consisted of 100mM KH_2_PO_4_/K_2_HPO_4_ (Fisher) with 4mM tetrabutylammonium-bisulfate (Sigma) in water. Buffer B consisted of 100mM KH_2_PO_4_/K_2_HPO_4_ with 4mM tetrabutylammonium-bisulfate in 30% methanol (Fisher Scientific). After 2.5 min at 0% buffer B, the gradient profile started with a linear increase of buffer B to 30% until 5.0 min, followed by a linear increase to 50% buffer B until 10.0 min. From 10.0 min to 18.0 min the gradient was increased linearly to 100% buffer B. At 20.0 min, the gradient was reversed to 0% buffer B and held for 5.0 min. Detection was achieved by measuring absorbance at 254nm and quantified against the absorbance of known standards.

#### Seahorse XF Glycolytic and Oxidative Stress Tests

An XF24-3 Analyzer (Agilent) equipped with fluorescent biosensors was used to measure local pH or pO_2_ changes in culture media. Briefly, 50 000 MEFs were seeded per culture well overnight (8-12h), then washed and equilibrated in glucose/pyruvate-free, unbuffered (glycolysis stress test) or low glucose/low pyruvate, HEPES-free DMEM for 1h at 37°C, in a CO_2_-free atmosphere. After taking baseline recordings, reagents were injected successively into each well, with pH and pO_2_ tracked in real-time (2min-1min-2min / mix-wait-measure cycles, with three cycles per injection). Working concentrations optimized for the “glycolytic stress test” were 10mM glucose, 2.5μM oligomycin and 12mM 2-deoxyglucose. Those used for the “mitochondrial stress test" were 10mM glucose, 0.8μM oligomycin, 1.2μM FCCP and a 4μM antimycin/2μM rotenone mixture. After the final reading, live cells were stained with calcein AM dye (Life Technologies), and each well imaged for particle analysis in ImageJ. Extracellular acidification rates and oxygen consumption rates for each well were divided by the live cell counts for the same well, and the final result normalized to “50 000 viable cells at the end of the experiment”.

To measure fatty acid oxidation, cells were first starved over 24h in glucose/pyruvate-limited DMEM supplemented with 0.5mM carnitine. Thereafter, cells were washed and kept briefly in Krebs-Henseleit Buffer. Either BSA-conjugated palmitate or BSA vehicle was added to wells, and the oxidative stress test was immediately run. Fatty acid oxidation was taken to be the difference between palmitate and vehicle-treated readouts. Separate control wells treated with 40μM etomoxir 15min before the experiment were found to be unresponsive to palmitate – confirming that oxidation measured in this setup was indeed attributable to fatty acids.

To acquire similar readouts under hypoxic conditions, the XF24-3 Analyzer (Agilent) was subsequently placed within a gas flow-controlled Perspex hermetic chamber. The chamber was not humidity- or temperature-controlled, but a fan promoted internal air circulation. The chamber was maintained at 1% oxygen, 0% carbon dioxide. The assay was optimized and cells were seeded at a density that allowed anoxia to be avoided during each measurement cycle. The instrument and biosensors were allowed to equilibrate in this hypoxic atmosphere overnight, while aliquots of assay reagents were thawed and equilibrated 1h in advance.

MEFs (20 000) were seeded per well, and cultured for 24h at 1% oxygen, 5% carbon dioxide. After cells were washed and left in XF media, they were transferred into the Analyzer via an airtight container, and were left to equilibrate with the instrument’s carbon dioxide-free atmosphere for 1h. The glycolytic and mitochondrial stress tests were performed as previously described. Reagent working concentrations were modified as follows: For the “glycolytic stress test”, 10mM glucose, 2.5μM oligomycin and 12mM 2-deoxyglucose. For the “mitochondrial stress test", 10mM glucose, 0.6μM oligomycin, 1μM FCCP and a 2μM antimycin/1μM rotenone mixture.

To analyze data acquired under hypoxia, extracellular acidification rates did not require further correction. Oxygen consumption rates were corrected using the XF Hypoxia Rate Calculator Program (Seahorse Bioscience), which was calibrated by wells where anoxia was ‘induced’ by repeated injections of 100mM Na_2_SO_3_ into XF assay calibrant. Well readings were finally normalized to viable cell counts performed at the end of each experiment, taken to be the number of cells stained positively by calcein AM dye (Life Technologies).

#### Western Blotting of Cells and Organ Lysates

Confluent layers of cultured cells were washed once with DPBS and scraped into ice-cold RIPA buffer (1% PMSF, 2% proteinase inhibitor) for protein extraction. Primary tissues were homogenized under liquid nitrogen with mortar and pestle, then protein was likewise extracted with a 1:1 tissue:RIPA volume ratio. Lysates (15μg) were separated on either 3-8% Tris-Acetate or 4-12% Bis-Tris gels (Invitrogen) in SDS buffer, then transferred onto nitrocellulose membranes with a TransBlot system (BioRad). Immunoblotting was performed by standard methods. A list of primary and secondary antibodies is appended in the Key Resources Table. Blots were developed with an Amersham ECL Western Blotting Detection Reagent (GE Healthcare) and visualized with a Fusion FX system (Vilber Lourmat). Densitometric analysis was performed with ImageJ.

#### Comparison of Expression of Mitochondrial Electron Transfer Chain Complexes in WT and FIH KO

MEFs seeded in six-well plates were lysed in 100uL/well RIPA buffer. Lysates were separated on 4-12% SDS Page, transferred to nitrocellulose membrane. Immunoblotting was performed with a Total OXPHOS Rodent WB Antibody Cocktail (Abcam 110413) 1:250, using rat heart mitochondria as a positive control.

#### Tail Vein Blood Sampling

Serum obtained from tail vein blood samples was analysed by a biochemical core facility using the following assays (Dade-Behring): creatine kinase by Oliver and Rosalki method; glucose by hexose-6-phosphate dehydrogenase assay; high density lipoprotein by homogenous detergent solubility / enzyme assay; lactate by Marbech and Weil method.

#### Indirect Calorimetry

Mouse energy expenditure was measured with an Oxymax system (Columbus Instruments). Each mouse was placed in a metabolic chamber with a controlled supply of room air and monitored over a period of 24h, during which it was provided freely with food and water. Each pair of wildtype and knockout littermates was analyzed during the same 24h timeframe. O_2_ and CO_2_ % at each chamber inlet and outlet were monitored with a paramagnetic O_2_ sensor and CO_2_ sensor and analyzed to obtain weight-normalized metabolic parameters (VO_2_, VCO_2_ and respiratory exchange ratios) over time.

#### Incremental Uphill and Downhill Treadmill Protocols

All calorimetry and treadmill studies were carried out at a controlled 21 degree C ambient environmental temperature. No exclusion or inclusion criteria were employed when selecting mice in these studies.

Untrained knockout and control mouse littermates were run on an enclosed-chamber modular treadmill (Columbus Instruments) at a 5° incline, beginning at 5m/min, with an acceleration of 1m/min^2^ until the animal was exhausted, exhaustion being defined as the point where mice refused to run even when coming into contact with a low-voltage power grid. Oxygen and carbon dioxide into and out of the treadmill chamber was monitored using a carbon dioxide and paramagnetic oxygen sensor within an Oxymax system. VO_2,max_ was defined as the maximum VO_2_ achieved during this assessment, while the baseline VO_2_ was taken to be that at rest, just before the treadmill was started at 5m/min. Power was calculated as the product of bodyweight in kg, acceleration due to gravity, and vertical speed (horizontal speed × angle of incline). To assess downhill running, the VO_2,max_ protocol was repeated, with the treadmill positioned at a 10° decline instead.

To assess the impact of inhalational hypoxia on running performance, mice were first allowed to acclimatize for 5h in hermetic chambers supplied at 12% oxygen. They were then transferred to the modular treadmill setup (Columbus Instruments) with a joint gas supply, and the VO_2,max_ protocol was repeated.

To assess endurance, with at least 72h rest between any two protocols, mice were run at 70% of their pre-determined, maximum achievable speed at a 5° incline. Distance run to exhaustion was noted. To measure oxygen debt, from a resting state, mice were immediately stepped up to 70% of their pre-determined, maximum achievable speed at a 5° incline, run for 15min, then immediately stepped down to rest. The resulting plot of VO_2_ over time was used for analysis. Oxygen debt was defined as the area under the VO_2_ curve following the ‘step-down’.

#### Doppler Imaging

Procedures were carried out at a 21 degree C environmental temperature. Mice were anaesthetized with isoflurane atop a warm platform, and the hindlimb was exposed by a skin flap. Doppler imaging was performed with a laser speckle contrast imager (FLPI, Moor Instruments). The charged coupled device camera was positioned 20cm above the exposed femoral artery and its branches. Four sequential images were acquired in a in a long exposure, high resolution setting (camera exposure 1min), integrated to produce a scaled flux image of local blood flow velocity. Perfusion was reported in arbitrary units. Three regions of interest across the femoral artery were analyzed and the data pooled. Imaging and analysis was done in a blinded fashion. Mice were culled at the end of the procedure by cervical dislocation.

#### Tissue Harvest

At the end of the experimental period, mice were culled by cervical dislocation. Samples of gastrocnemius, quadriceps and soleus were immediately snap-frozen in liquid nitrogen, while some samples were prepared for histology as described below.

#### Native Enzyme Activity Assays

Tissue was homogenized under liquid nitrogen using a mortar and pestle setup. Tissue homogenates were lysed in approximately 1:1 (v/v) non-denaturing Cell M Lysis Reagent (Sigma) containing proteinase inhibitor. Lysates were centrifuged, and supernatants kept at -80°C until analysis. Native citrate synthase activity (Sigma, CS0720) and cytochrome c oxidase activity (BioVision, K287-100) in lysates was measured using colorimetric kinetic measurements using a Tecan microplate reader.

#### Preparation of Tissue for Histology

For muscle histology, samples of quadriceps were harvested from untrained mice of all genotypes and flash-frozen in OCT (Tissue-Tek) by immersing in a dewar of liquid nitrogen-cooled isopentane. Tissue sections (10μm) were cut with a cryostat for histological analysis.

#### Fiber Type Differentiation Using NADH-tetrazolium Reductase Method

Frozen, unfixed mouse quadriceps sections were incubated for 30min in 0.16% NADH in 0.05M Tris buffer (pH7.6) containing freshly-added 0.1% nitro-blue tetrazolium (Sigma), at 37°C. Sections were washed with three exchanges of deionised water. Unbound nitro-blue tetrazolium was removed using acetone solutions in the following sequence: 30%, 60%, 90%, 60%, 30%. Finally, slides were rinsed several times with deionized water and cover-slipped with an aqueous mounting medium. Purple formazan is deposited at sites of mitochondria; more oxidative fibers appear darker, as do blood vessels. The segmentation function of the MRI Adipocyte Tools toolset in ImageJ was used to demarcate and enumerate individual myofibers of a minimum surface area per field. The staining intensities of individual fibers were then stratified into three grades using a LUT editor, and the fibers belonging to each grade were then manually counted by two blinded observers, finally expressed as a ratio of total myofibers per field.

#### Metabolite Extraction from Muscle Samples

Gastrocnemius samples of known mass were homogenized under liquid nitrogen using a mortar and pestle setup. 100ul of each homogenate was added to 300μl of ice-cold methanol:chloroform (2:1) and vortexed. Mixtures were kept on ice for 15min, then 100ul of chloroform and 100μl of water were added to each tube. Samples were centrifuged for 7min at 13 300rpm, then the lower (lipophilic) fraction was carefully separated and dried in a fume hood overnight, then stored at -80°C until analysis.

#### LC-MS Analysis of Lipid Species

75% of dried lipophilic fractions were used for liquid chromatography-mass spectrometry (LC-MS) analysis of intact lipids. Each extract was reconstituted in 300 μl isopropanol-acetonitrile-water (2:1:1 v/v), and then 100 μl of this was diluted in 1.4ml IPA-acetonitrile-water. Samples were then analysed using a Waters Q-ToF Xevo (Waters Corporation, Manchester) combined with an Acquity Ultra Performance Liquid Chromatogram (UPLC). 4 μl of the sample was injected onto an Acquity UPLC Charged Surface Hybrid C18 column (1.7 μm x 2.1 mm x 100 mm) (Waters Corporation, Manchester, UK) held at 55°C. The binary solvent system (flow rate 0.400 ml/min) consisted of solvent A containing HPLC grade acetonitrile-water (60:40) with 10 mM ammonium formate and solvent B consisting of LC-MS grade acetonitrile-isopropanol (10:90) and 10 mM ammonium formate. The gradient started from 60% A/40% B, reached 99% B in 18 min, then returned back to the starting condition, and remained there for the next 2 min. The data was collected over the mass range of *m/z* 105-1800 with a scan duration of 0.2s. The source temperature was set at 120°C and nitrogen was used as the desolvation gas (900 L/h). The voltages of the sampling cone, extraction cone and capillary were 30 kV, 3.5 kV and 2 kV respectively, with a collision energy of 6 V for each single scan, and a collision ramp from 20 to 40 V for the fragmentation function. As lockmass, a solution of 2 ng/μl acetonitrile-water (50:50) leucine enkephaline (*m/z* 556.2771) with 0.1% formic acid was infused into the instrument every 30 seconds.

### Quantification and Statistical Analysis

Analyses were performed with GraphPad Prism 7. Data are presented as mean ± SD. All experiments were conducted at least three times with representative data shown, and where appropriate, examined for normal distribution by histogram. Statistically significant differences were determined using the Student’s t test. Correlations were evaluated using the Pearson r method. A value of p <0.05 was considered statistically significant. For the analyses of metabolites, comparisons between groups were made using multiple t tests with a false discovery rate of 0.05. For comparison among multiple groups a, one-way ANOVA was used; for comparison of grouped data, a two-way ANOVA was used. Statistical parameters can be found in the figure legends.

## Supplemental Information

Document S1. Figures S1–S5 and Tables S1–S3

Document S2. Article plus Supplemental Information
