# Supplementary material for: The Factor Inhibiting HIF Asparaginyl Hydroxylase Regulates Oxidative Metabolism and Accelerates Metabolic Adaptation to Hypoxia
Source: Cell Metab. 2018 Apr 3;27(4):898–913.e7. doi: 10.1016/j.cmet.2018.02.020 (PMC5887987; doi:10.1016/j.cmet.2018.02.020)
Supplement: Document S1. Figures S1–S5 and Tables S1–S3 [file mmc1.pdf]

Cell Metabolism, Volume *27*

Supplemental Information

The Factor Inhibiting HIF Asparaginyl Hydroxylase Regulates Oxidative Metabolism and Accelerates Metabolic Adaptation to Hypoxia

Jingwei Sim, Andrew S. Cowburn, Asis Palazon, Basetti Madhu, Petros A. Tyrakis, David Macías, David M. Bargiela, Sandra Pietsch, Michael Gralla, Colin E. Evans, Thaksaon Kittipassorn, Yu C.J. Chey, Cristina M. Branco, Helene Rundqvist, Daniel J. Peet, and Randall S. Johnson

Supplemental Figure Legends

Figure S1 Genetic deletion of FIH and/or VHL using a cre-lox system. Related to Figure 1.

A. Immunoblots of whole cell lysates.

Figure S2 Further metabolic characterization of FIH and/or VHL null cells. Related to Figure 2.

A. Culture media lactate levels. Mouse embryonic fibroblast (MEF) genotypes as specified.
B. Culture media glucose levels. MEF genotypes as specified.
C. Flow cytometry; representative histograms of cell count versus MitoSOX (superoxide stain) fluorescence, with shaded graph representing unstained controls. Three experiments were performed with two biological replicates each; the histogram provided is representative of one of these experiments.
D. Flow cytometry; MitoSOX fluorescence as before, but of cells after two days of culture in a hypoxic chamber.
E. Flow cytometry; representative histograms of cell count versus TMRM fluorescence (indicator of mitochondrial membrane potential), with shaded graph representing unstained controls.
F. Flow cytometry; TMRM fluorescence as before, but of cells after two days of culture in a hypoxic chamber.

Figure S3 Rescue of oxidative phenotype in FIH null cells with FIH overexpression. Related to Figure 4.

A. Immunoblot of whole cell extracts from $FIH^{-/-}$ MEF parental cell line stably transfected with empty vector (CTRL), FIH parental cell line stably overexpressing FIH (OE FIH), parental $FIH^{-/-}$ line and the $FIH^{fl/fl}$ line probed for FIH and α-Paxillin. Black line indicates non-adjacent lanes on the same immunoblot.
B. Oxidative stress test performed on the $FIH^{-/-}$ MEF parental cell line stably transfected with empty vector (CTRL) and the FIH parental cell line stably overexpressing FIH (OE FIH), using a Seahorse XFe96 Analyzer. Cells were seeded in an XF96 cell culture microplate at 10,000 cells per well overnight, and then washed, equilibrated and assayed in XF base media with 10 mM glucose and 1 mM pyruvate. The assay was performed using 0.8 µM oligomycin, 1.2 µM FCCP, and a mixture of 2 µM rotenone and 2 µM antimycin A. Cells were stained with NucBlue Live ReadyProbes reagent (Thermo Fisher Scientific) and each well imaged for particle analysis in ImageJ. OCR data are presented as mean ± SEM. *n* = 6 independent cell culture samples per genotype.

Figure S4 Further metabolic characterization of FIH and/or VHL null skeletal muscle mutant mice. Related to Figure 5.

A. Immunoblots of whole muscle lysates showing successful, global deletion of FIH in FIH nullizygous mice, striated muscle-specific deletion of FIH using muscle

creatine kinase (CKMM)-cre, and skeletal muscle-specific deletion of VHL and/or FIH using Myf6-cre.

B. 24h indirect calorimetry readouts showing baseline VO_2 values of unrestrained mice, with dark (shaded in grey) and light (unshaded) photoperiods demarcated.
C. 24h indirect calorimetry readouts showing baseline VCO_2 values of unrestrained mice, with dark (shaded in grey) and light (unshaded) photoperiods demarcated.
D. VHL muscle-specific mutant mice. 24h indirect calorimetry readouts (baseline VO_2 values) in dark and light photoperiods.
E. VHL muscle-specific mutant mice. 24h indirect calorimetry readouts (baseline VCO_2 values) in dark and light photoperiods.
F. Relationship between respiratory exchange ratio (RER) and uphill running speed, graphed for muscle-specific mutant mice of various genotypes and their respective control mice. For statistical analysis, a linear fit was assumed, and the best-fit equations were compared. n=7 per group.
G. Fiber type composition in cross-sections of deep quadriceps, based on fiber type-specific staining. Loss of VHL/FIH decreases the percentage of type IIb glycolytic fibers.

Figure S5 Further metabolic characterization of FIH-null skeletal muscle. Related to Figure 6.

A. Running uphill to exhaustion increases circulating blood creatine kinase in the FIH muscle-specific mutant, while blunting the high circulating HDL levels seen in knockout mice at rest. Blood glucose is not affected.
B. Principal component analysis comparing the lipophilic metabolomes of gastrocnemius samples from FIH muscle-specific mutant and control mice, at rest and after running uphill to exhaustion (“postex”). A list of metabolites best distinguishing the genotypes was determined for each condition (rest versus exercise), and filtered by $p<0.05$ (resulting in 35 metabolites at rest, and 50 metabolites post-exercise), then parsed into lipid metabolite classes by their retention times. The composition of each series of "top hits" has been schematized and presented here.
C. FIH muscle-specific mutants’ RERs showed greater separation from control RERs ($p=0.0005$) at high speeds of downhill running. For statistical analysis, a linear fit was assumed, and the best-fit equations were compared. n=7 per group.
D. Oxidative gene mRNA profiling from whole muscle lysates. n=5 mice per group.
E. Representative transmission electron microscopy images of FIH muscle-specific mutant soleus and gastrocnemius samples showing no gross distortions in mitochondrial structure and distribution. Scale bar representative of 2 microns.

** p<0.05, ** p<0.01, *** p<0.001. Data are represented as mean ± SEM. For multiple comparisons, a one-way ANOVA was used. For pairwise comparisons, a two-tailed student’s t-test was used. For in vitro experiments, n=3 independent cell culture samples per group. For in vivo experiments, only male mice were used, with male littermates as controls. n=7 mice/genotype.*

Figure S1

A

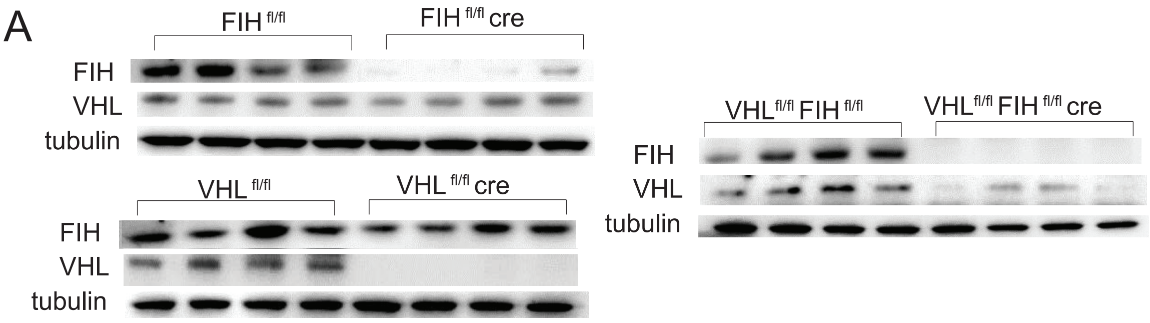
FIH fl/fl
FIH fl/fl cre
FIH
VHL
tubulin
VHL fl/fl
VHL fl/fl cre
FIH
VHL
tubulin
VHL fl/fl FIH fl/fl
VHL fl/fl FIH fl/fl cre
FIH
VHL
tubulin

Figure S2

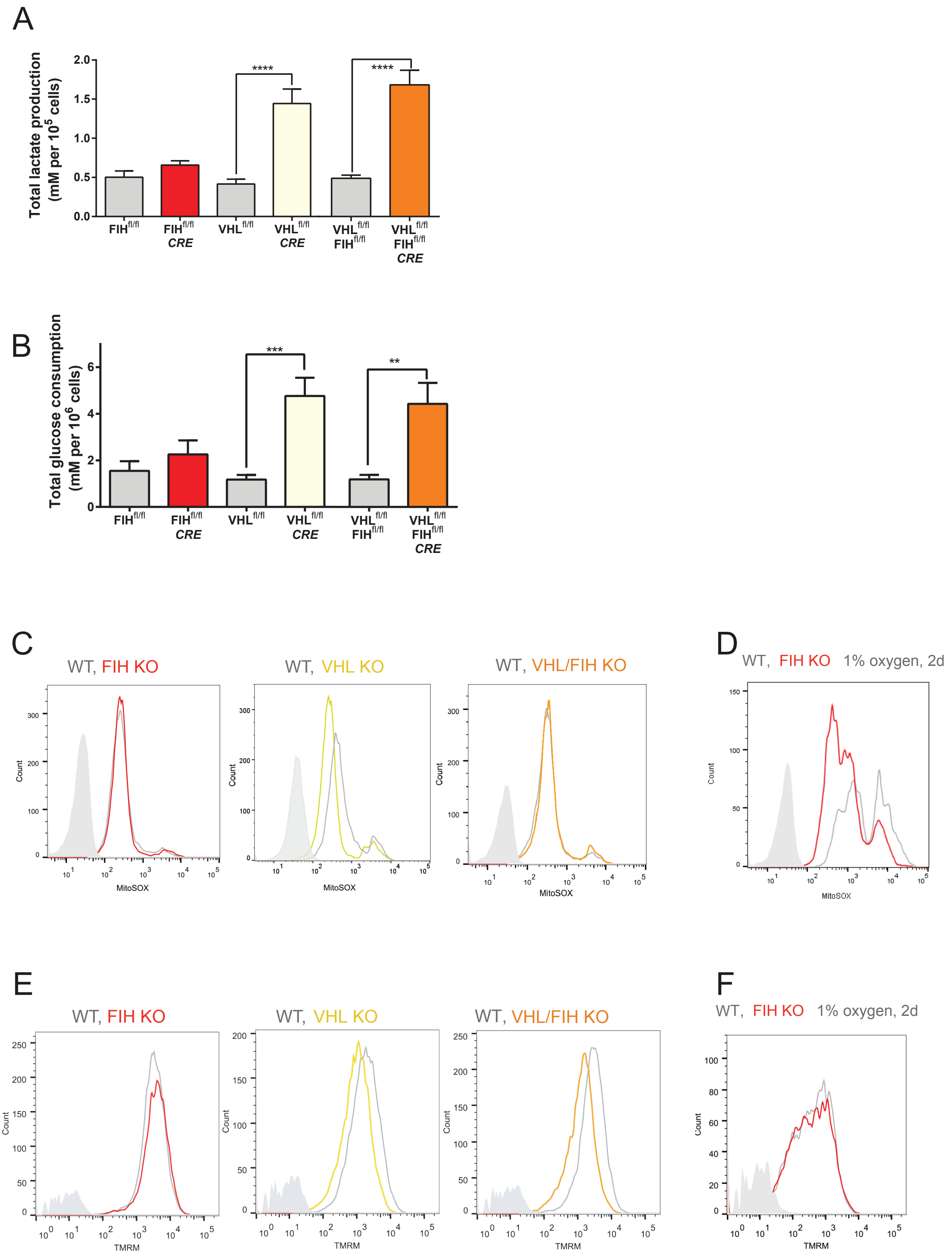
A
Total lactate production (mM per 10^5 cells)
2.0
1.5
1.0
0.5
0.0
****
****
FIH fl/fl
FIH fl/fl CRE
VHL fl/fl
VHL fl/fl CRE
VHL fl/fl FIH fl/fl
VHL fl/fl FIH fl/fl CRE
B
Total glucose consumption (mM per 10^6 cells)
6
4
2
0
***
**
FIH fl/fl
FIH fl/fl CRE
VHL fl/fl
VHL fl/fl CRE
VHL fl/fl FIH fl/fl
VHL fl/fl FIH fl/fl CRE
C
WT, FIH KO
WT, VHL KO
WT, VHL/FIH KO
Count
MitoSOX
D
WT, FIH KO 1% oxygen, 2d
Count
MitoSOX
E
WT, FIH KO
WT, VHL KO
WT, VHL/FIH KO
Count
TMRM
F
WT, FIH KO 1% oxygen, 2d
Count
TMRM

Figure S3

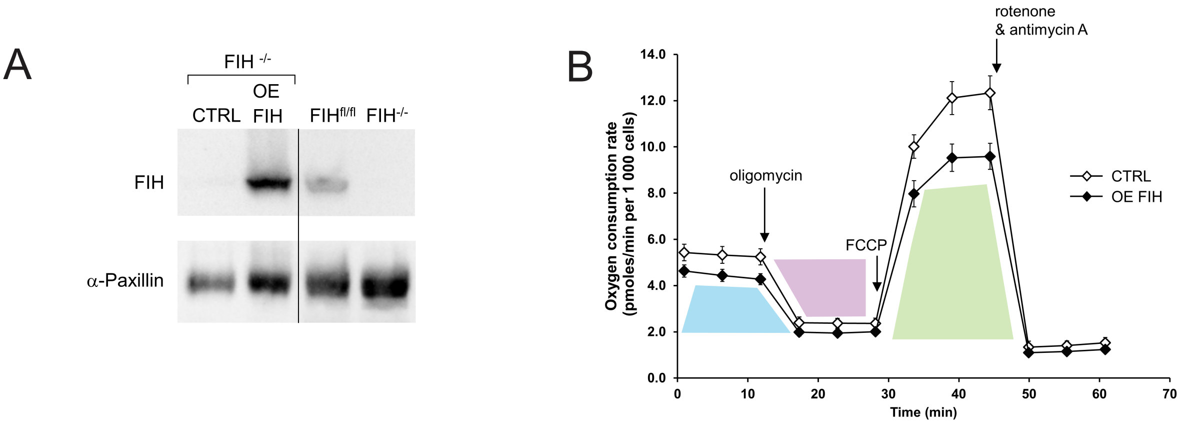
A
FIH -/-
CTRL
OE FIH
FIH$^{fl/fl}$
FIH$^{-/-}$
FIH
α-Paxillin
B
rotenone & antimycin A
oligomycin
FCCP
CTRL
OE FIH
Oxygen consumption rate (pmoles/min per 1 000 cells)
Time (min)
14.0
12.0
10.0
8.0
6.0
4.0
2.0
0.0
0
10
20
30
40
50
60
70

Figure S4

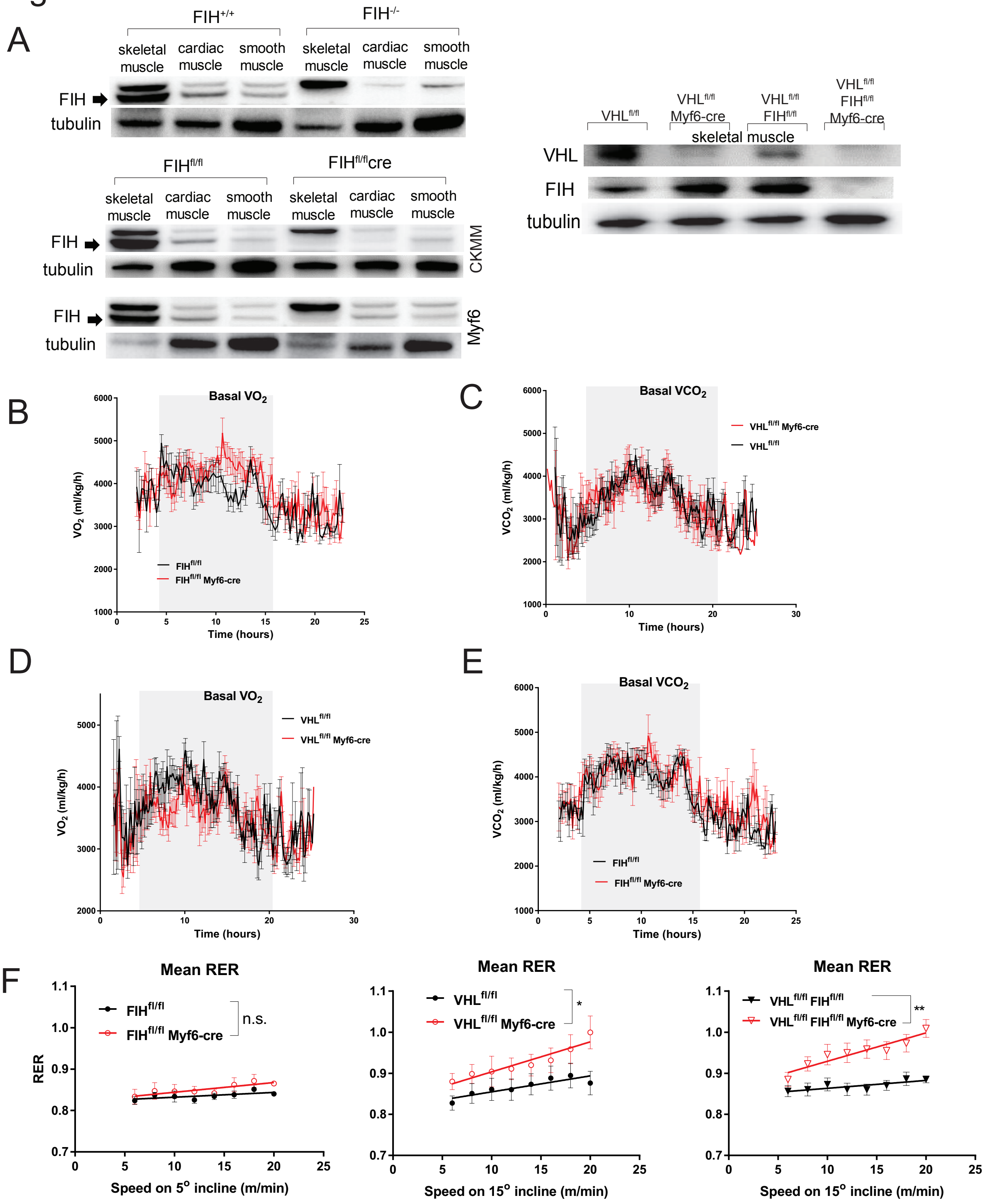

G

	Fibre type (%)	FIH fl/fl	FIH fl/fl Myf6-cre	VHL fl/fl	VHL fl/fl Myf6-cre	VHL fl/fl FIH fl/fl	VHL fl/fl FIH fl/fl Myf6-cre
Deep quadriceps	Type I	14.8 ± 2.3	15.5 ± 2.6	20.0 ± 3.8	16.0 ± 2.7	15.2 ± 3.9	25.7 ± 1.8
	Type IIA	35.8 ± 2.1	35.5 ± 1.9	30.7 ± 1.8	30.5 ± 2.5	34.9 ± 2.5	33.0 ± 1.5
	Type IIB	49.6 ± 1.4	48.9 ± 2.3	49.0 ± 3.8	53.6 ± 2.0	50.5 ± 2.4	40.3 ± 2.0 **

Figure S5

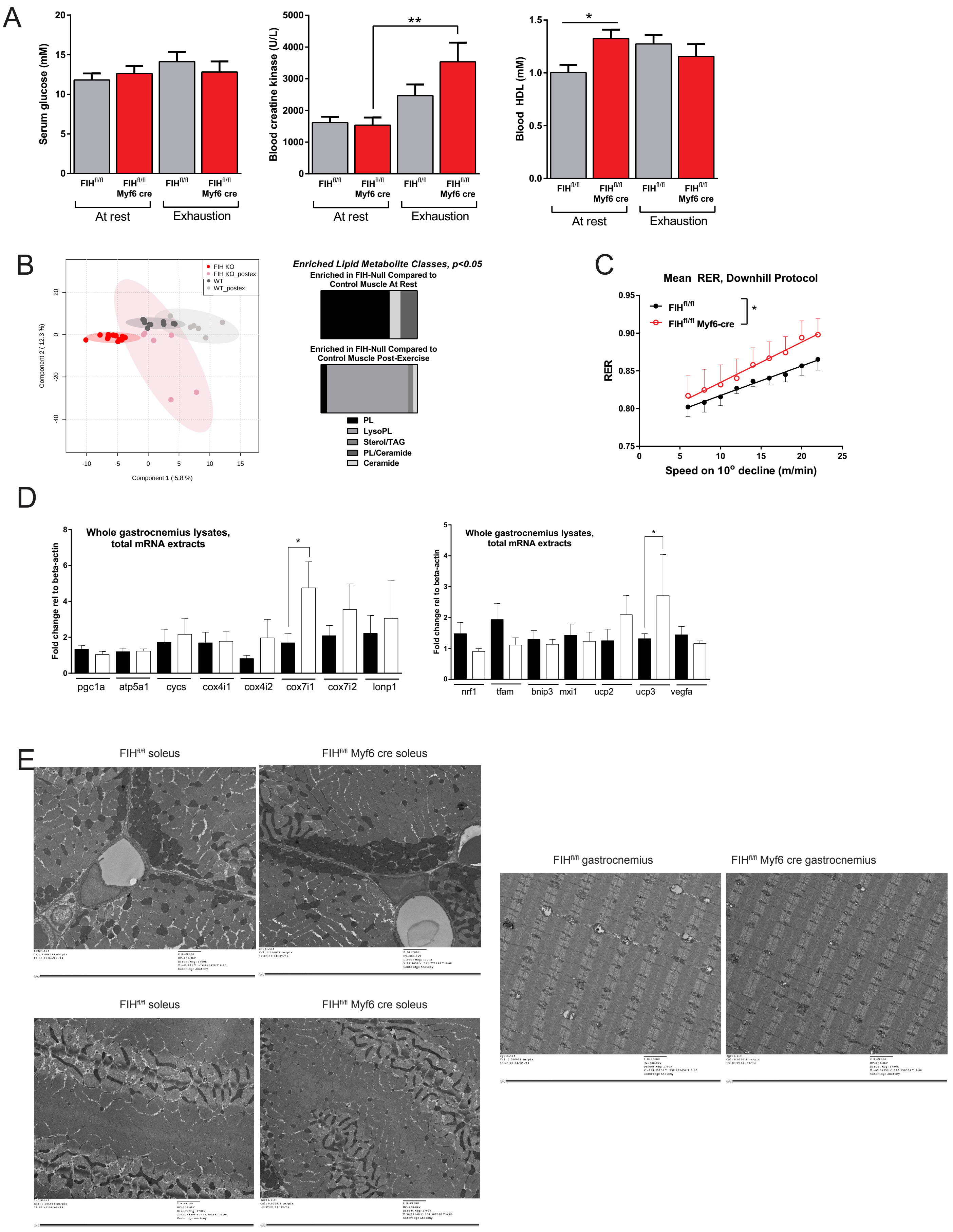
A
Serum glucose (mM)
FIH fl/fl
FIH fl/fl Myf6 cre
At rest
Exhaustion
Blood creatine kinase (U/L)
**
Blood HDL (mM)
*
B
FIH KO
FIH KO_postex
WT
WT_postex
Component 1 (5.8 %)
Component 2 (12.3 %)
Enriched Lipid Metabolite Classes, p<0.05
Enriched in FIH-Null Compared to Control Muscle At Rest
Enriched in FIH-Null Compared to Control Muscle Post-Exercise
PL
LysoPL
Sterol/TAG
PL/Ceramide
Ceramide
C
Mean RER, Downhill Protocol
FIH fl/fl
FIH fl/fl Myf6-cre
RER
Speed on 10° decline (m/min)
D
Whole gastrocnemius lysates, total mRNA extracts
Fold change rel to beta-actin
pgc1a
atp5a1
cycs
cox4i1
cox4i2
cox7i1
cox7i2
lonp1
nrf1
tfam
bnip3
mxi1
ucp2
ucp3
vegfa
E
FIH fl/fl soleus
FIH fl/fl Myf6 cre soleus
FIH fl/fl gastrocnemius
FIH fl/fl Myf6 cre gastrocnemius

Table S1 qPCR primers. Related to Methods.

12S (mtDNA) rRNA forward	ACCGCGGTCATACGATTAAC
12S (mtDNA) rRNA reverse	CCCAGTTTGGGTCTTAGCTG
18S rRNA forward	CGGCGACGACCCATTCGAAC
18S rRNA reverse	GAATCGAACCCTGATTCCC
18S rDNA forward	TAGAGGGACAAGTGGCGTTC
18S rDNA reverse	CGCTGAGCCAGTCAGTGT
Ca9 forward	CCTCTCCCGGAACTGAGCCTAT
Ca9 reverse	TGTTCTGAGCCTGGGTGATCTG
catalase forward	AGCGACCAGATGAAGCAGTG
catalase reverse	TCCGCTCTCTGTCAAAGTGTG
Cox4i1 forward	ATTGGCAAGAGAGCCATTTCTAC
Cox4i1 reverse	CACGCCGATCAGCGTAAGT
Cox4i2 forward	CTGCCCGGAGTCTGGTAATG
Cox4i2 reverse	CAGTCAACGTAGGGGGTCATC
Cox7a1 forward	GCTCTGGTCCGGTCTTTTAGC
Cox7a1 reverse	GTACTGGGAGGTCATTGTCGG
Cox7a2 forward	ATCAGCACCACTTCACGAAGG
Cox7a2 reverse	GAACTGGCATCCCATTATCCTC
Cyt b forward	GCTTTCCACTTCATCTTACCATTTA
Cyt b reverse	TGTTGGGTTGTTTGATCCTG
Cyt c forward	CCAAATCTCCACGGTCTGTTC
Cyt c reverse	ATCAGGGTATCCTCTCCCCAG
Eno1 forward	TGCGTCCACTGGCATCTAC
Eno1reverse	CAGAGCAGGCGCAATAGTTTTA
Fih forward	GTGCCAGCACCCATAAGTT
Fih reverse	CGCGCTGCTGTATAGCTT
Gapdh forward	ACCACAGTCCATGCCATCAC
Gapdh reverse	TCCACCACCCTGTTGCTGTA
Glut1 forward	GGGCATGTGCTTCCAGTATGT
Glut1reverse	ACGAGGAGCACCGTGAAGAT
Gpi forward	TCAAGCTGCGCGAACTTTTTG
Gpi reverse	GGTTCTTGGAGTAGTCCACCAG
HkII forward	TGATCGCCTGCTTATTCACGG
HkII reverse	AACCGCCTAGAAATCTCCAGA
Ldha forward	TGTCTCCAGCAAAGACTACTGT
Ldha reverse	GACTGTACTTGACAATGTTGGGA
Lonp1 forward	CGGGAAGATCATCCAGTGTT
Lonp1 reverse	ACGTCCAGGTAGTGGTCCAG
M5s_rDNA forward	ACCGTCTAGCCGTCCTCCTT
M5s_rDNA reverse	CCCACTGAGGATGGATACATG
Mcd fwd	GCACGTCCGGGAAATGAAC

Mcd rev	GCCTCACACTCGCTGATCTT
Mco1 forward	CCCAGATATAGCATTCCCACGA
Mco1 reverse	AGCAAGCTCGTGTGTCTACATC
Mct1 forward	TGTTAGTCGGAGCCTTCATTTC
Mct1 reverse	CACTGGTCGTTGCACTGAATA
Mct4 forward	TCACGGGTTTCTCCTACGC
Mct4 reverse	GCCAAAGCGGTTCACACAC
Mxi1 forward	GATTCAGAGCGAGAGGAGATTG
Mxi1reverse	ACACTGGCACTGGAGTAACC
Pdk1 forward	GAAGCAGTTCCTGGACTTCG
Pdk1 reverse	CCAACTTTGCACCAGCTGTA
Pgc1α forward	TATGGAGTGACATAGAGTGTGCT
Pgc1α reverse	CCACTTCAATCCACCCAGAAAG
Pgk forward	CTGTGGTACTGAGAGCAGCAAGA
Pgk reverse	CAGGACCATTCCAAACAATCTG
Pkm forward	GCCGCCTGGACATTGACTC
Pkm reverse	CCATGAGAGAAATTCAGCCGAG
Sod1 forward	AACCAGTTGTGTTGTCAGGAC
Sod1 reverse	CCACCATGTTTCTTAGAGTGAGG
Sod2 forward	CAGACCTGCCTTACGACTATGG
Sod2 reverse	CTCGGTGGCGTTGAGATTGTT
Tpi forward	CCAGGAAGTTCTTCGTTGGGG
Tpi reverse	CAAAGTCGATGTAAGCGGTGG

Table S2 Extracellular metabolites distinguishing FIH KO over control MEF media, from ^{1}H-NMR analysis. Related to Figure 1. A positive t.stat means that a particular metabolite is upregulated in FIH KO compared to control MEFs, whereas a negative t.stat means it is downregulated.

	t.stat	p value	FDR
Alanine	18.962	5.71E-21	1.31E-19
Formate	12.42	5.98E-15	6.88E-14
Glutamine	-10.17	2.14E-12	1.64E-11
Lactate	6.1856	3.17E-07	1.82E-06
Glucose	-5.9558	6.55E-07	3.01E-06
Pyruvate	4.7558	2.84E-05	0.00010881
Choline	-4.5858	4.80E-05	0.00015583
Isoleucine	-4.5461	5.42E-05	0.00015583
Leucine	-3.9729	0.00030602	0.00078204
Pyroglutamate	-2.4035	0.021227	0.048823

Table S3 Extracellular metabolites distinguishing VHL KO over control MEF media, ^{1}H-NMR analysis. Related to Figure 1. A positive t.stat means that a particular metabolite is upregulated in VHL KO compared to control MEFs, whereas a negative t.stat means it is downregulated.

	t.stat	p value	FDR
Lactate	19.199	1.80E-12	2.98E-11
Formate	18.744	2.60E-12	2.98E-11
Pyruvate	-11.247	5.23E-09	4.01E-08
Glutamine	8.3394	3.22E-07	1.85E-06
Alanine	7.1587	2.27E-06	1.05E-05
Glycine	5.5381	4.50E-05	0.00017242
Leucine	5.0049	0.00012959	0.00037663
Choline	4.9995	0.000131	0.00037663
Glucose	4.822	0.00018773	0.00047974
Acetate	-4.6787	0.00025158	0.00057864
Methionine	3.1095	0.0067443	0.014102
Threonine	-2.7998	0.012846	0.024622
Isoleucine	2.7013	0.01573	0.02783
Arginine	2.6512	0.017426	0.028629
